# Rapamycin treatment dose‐dependently improves the cystic kidney in a new ADPKD mouse model *via* the mTORC1 and cell‐cycle‐associated CDK1/cyclin axis

**DOI:** 10.1111/jcmm.13091

**Published:** 2017-02-28

**Authors:** Ao Li, Song Fan, Yuchen Xu, Jialin Meng, Xufeng Shen, Jun Mao, Li Zhang, Xiansheng Zhang, Gilbert Moeckel, Dianqing Wu, Guanqing Wu, Chaozhao Liang

**Affiliations:** ^1^ Department of Urology PKD Center The First Affiliated Hospital of Anhui Medical University Hefei Anhui Province China; ^2^ State Key Laboratory of Molecular Oncology Cancer Hospital and Institute Chinese Academy of Medical Sciences and Peking Union Medical College Beijing China; ^3^ Department of Pathology Yale University School of Medicine New Haven CT USA; ^4^ Department of Pharmacology Yale University School of Medicine New Haven CT USA

**Keywords:** *Pkd2* mouse model, ADPKD, mTOR pathway, rapamycin

## Abstract

Although translational research into autosomal dominant polycystic kidney disease (ADPKD) and its pathogenesis has made considerable progress, there is presently lack of standardized animal model for preclinical trials. In this study, we developed an orthologous mouse model of human ADPKD by cross‐mating *Pkd2* conditional‐knockout mice (*Pkd2* ^f3^) to Cre transgenic mice in which Cre is driven by a spectrum of kidney‐related promoters. By systematically characterizing the mouse model, we found that *Pkd2* ^f3/f3^ mice with a Cre transgene driven by the mouse *villin‐*1 promoter (*Vil*‐Cre;*Pkd2* ^f3/f3^) develop overt cysts in the kidney, liver and pancreas and die of end‐stage renal disease (ESRD) at 4–6 months of age. To determine whether these *Vil*‐Cre;*Pkd2* ^f3/f3^ mice were suitable for preclinical trials, we treated the mice with the high‐dose mammalian target of rapamycin (mTOR) inhibitor rapamycin. High‐dose rapamycin significantly increased the lifespan, lowered the cystic index and kidney/body weight ratio and improved renal function in *Vil*‐Cre;*Pkd2* ^f3/f3^ mice in a time‐ and dose‐dependent manner. In addition, we further found that rapamycin arrested aberrant epithelial‐cell proliferation in the ADPKD kidney by down‐regulating the cell‐cycle‐associated cyclin‐dependent kinase 1 (CDK1) and cyclins, namely cyclin A, cyclin B, cyclin D1 and cyclin E, demonstrating a direct link between mTOR signalling changes and the polycystin‐2 dysfunction in cystogenesis. Our newly developed ADPKD model provides a practical platform for translating *in vivo* preclinical results into ADPKD therapies. The newly defined molecular mechanism by which rapamycin suppresses proliferation *via* inhibiting abnormally elevated CDK1 and cyclins offers clues to new molecular targets for ADPKD treatment.

## Introduction

Autosomal dominant polycystic kidney disease (ADPKD) is the most common of a group of inherited kidney disorders characterized by progressive cyst development and various extrarenal manifestations 1, 2. ADPKD, which has a worldwide prevalence estimated from 1:1000 to 1:4000, is the fourth most common single cause of end‐stage renal failure worldwide 3, 4, 5.

ADPKD, which primarily affects adults, is caused by mutations in the *PKD1* or *PKD2* genes, which encode the proteins polycystin‐1 (PC1) and polycystin‐2 (PC2), respectively. Approximately 85% of ADPKD patients have mutations in *PKD1*, and the remaining 15% have mutations in *PKD2* 6, 7. ADPKD follows a more severe course when it is caused by a *PKD1* mutation. The most common extrarenal manifestation of ADPKD is the formation of bile‐duct‐derived cysts in the liver 1, 8, 9, 10. Liver cysts occur in 83% of all ADPKD patients, and in 94% of those over 35 years of age 11, 12. Other ADPKD phenotypes include pancreatic cysts 13, 14, cerebral aneurysm 15, 16, 17, 18 and aortic root/thoracic aorta abnormalities 19, 20, 21.

There has been considerable progress in elucidating the molecular mechanisms and pathogenesis of ADPKD 3, 6, 22, 23, 24. However, there is no standardized animal model of ADPKD, and some non‐standardized ADPKD models are not systemically characterized. In addition, although several preclinical and clinical trials have reported that rapamycin has a beneficial effect on ADPKD 25, 26, no direct link between polycystin dysfunction and mTOR signalling changes or ADPKD cystogenesis has been established. In this study, we developed and well characterized a newly established ADPKD model and demonstrated that rapamycin inhibits cystic progression in the ADPKD kidney by down‐regulating the cell‐cycle‐associated CDK1 and cyclins (cyclin A, cyclin B, cyclin D1 and cyclin E), thereby arresting aberrant proliferation of the renal epithelia. The study not only generated a well‐characterized, standardized mouse model for ADPKD, but also revealed the involvement of the mTORC1–CDK1/cyclin axis in ADPKD, which leads to new molecular targets for treating this disease.

## Materials and methods

### Animals

This study was conducted using *Vil*‐Cre;*Pkd2* ^f3/f3^ mice and their normal *Pkd2* ^f3/f3^ littermates (control). All of the mouse models used in this study were from a *C57BL/6J* inbred background. *Pkd2* ^f3/f3^ mice 27 were bred with various Cre transgenic mice (Table 1) to generate *Pkd2* conditional‐knockout mice. Mouse genotyping and rapamycin treatment are detailed in Supplementary Methods A in Appendix S1.

**Table 1:** Cre transgenic mice used in this study

Nomenclature	Promoter	Exp. Time	Expressional Distribution	JAX Stock #	Ref.
γGt‐Cre	Rat gamma‐glutamyltransferase 1 promoter	E7.5	Cortical proximal tubules	012841	84 (Table S1)
*Nestin*‐Cre	Rat nestin promoter	E11.5	The central and peripheral nervous system, kidney, heart	003771	85 (Table S1)
*Villin*‐Cre	Mouse villin 1 promoter	E12.5	Embryonic intestinal endoderm adult intestinal epithelium vertical and horizontal axis	004586	30
Ksp‐Cre	Mouse cadherin 16 (*Cdh16* or Ksp‐cadherin) promoter	E15.5	Embryo: epithelial cells of developing nephrons, ureteric bud, mesonephric tubules, Wolffian duct, and Mullerian duct. Adult: renal tubules especially the collecting ducts, loops of Henle and distal tubules	012237	86 (Table S1)

### Cell lines, antibodies and reagents

The null‐*Pkd2* (E8) cell line and its maternally derived *Pkd2* heterozygous (D3) cell line were described in our previous study 27. Antibodies and reagents are listed in Supplementary Methods B in Appendix S1.

### Microarray analysis, quantitative PCR, histology, immunofluorescence (IF) and Western blots

Histology and IF staining procedures were as previously described 27, 28, 29. All other assays are described in Supplementary Methods C in Appendix S1.

### Statistics and measurements of cystic index, proliferation, apoptosis, blood urea nitrogen (BUN) and creatinine (Cr)

Statistics and detailed procedures of cystic index calculation, proliferation and apoptosis are described in Supplementary Methods D in Appendix S1.

## Results

### Development of a Pkd2 mouse model that mimics human ADPKD

**Figure 1:**
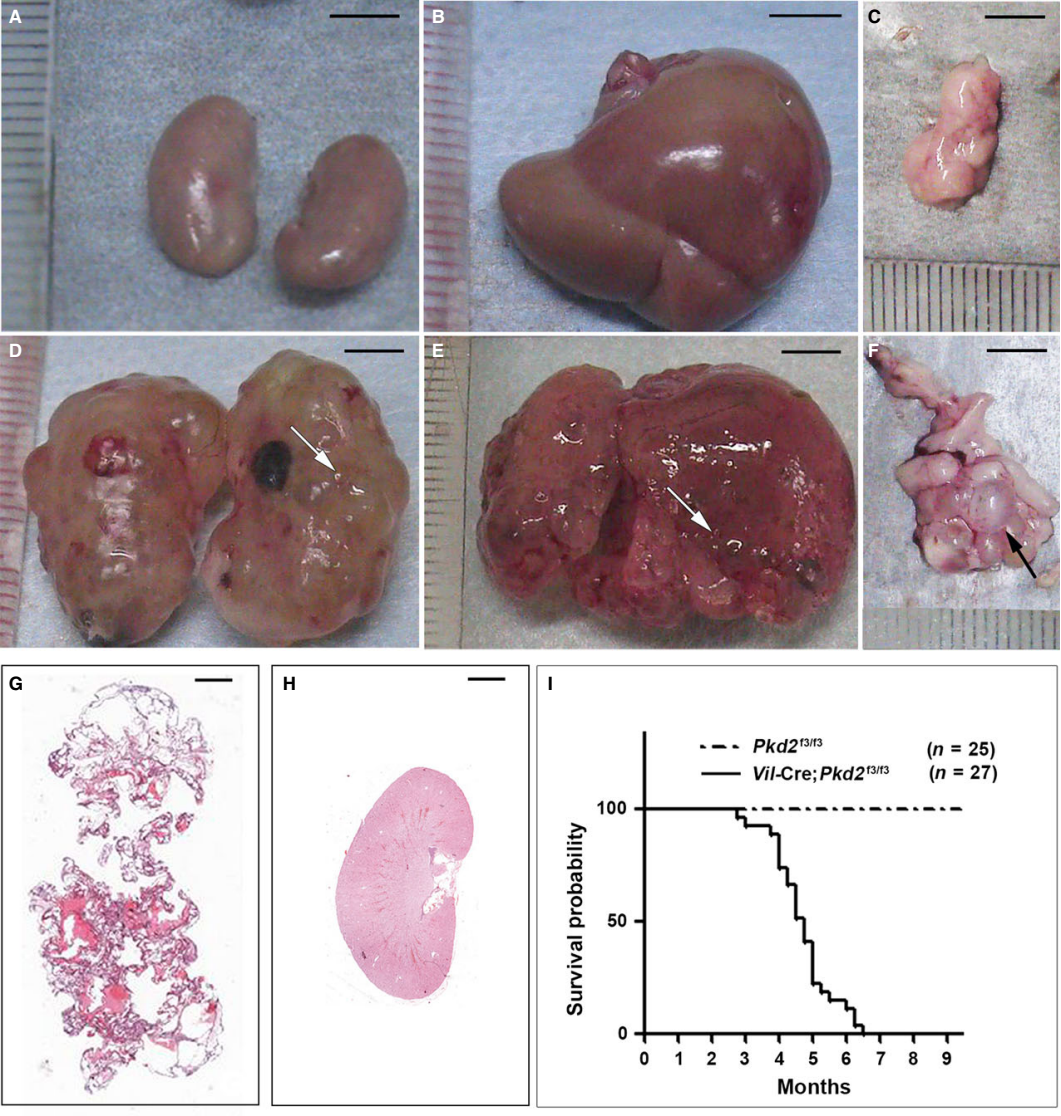
Mice with *Vil*‐Cre;*Pkd2* ^f3/f3^ alleles mimic the cystic phenotypes of human ADPKD. Compared to age‐matched control mice (**A**–**C**), 4‐month‐old *Vil*‐Cre;*Pkd2* ^f3/f3^ mice showed massive gross cysts (arrows) in the kidneys (**D**), liver (**E**) and pancreas (**F**). (**G**) H&E staining of a section from the kidney shown in (**D**). (**H**) The same H&E staining for the control kidney shown in (**A**). (**I**) Kaplan–Meier survival curves for *Vil*‐Cre;*Pkd2* ^f3/f3^ mice. The median survival time for *Vil*‐Cre;*Pkd2* ^f3/f3^ mice was about 4.5 months. Bars: 5 mm in **A**–**F**; 1 mm in **G** and **H**.

Standardized animal models for ADPKD would contribute greatly to the investigation of polycystin function, cystic pathogenesis and novel therapeutic compounds for treating ADPKD. Although we recently generated *Pkd2* conditional‐knockout mice (*Pkd2* ^f3/f3^) 27, we still needed to find an appropriate Cre‐recombination system by which the *Pkd2* ^f3/f3^ model could be used to represent the clinical manifestations of human ADPKD and to assess the effect of various therapeutic interventions. By cross‐mating *Pkd2* ^f3/f3^ mice with Cre transgenic mice driven by various kidney‐related promoters, including γ*Gt*‐Cre, Ksp‐Cre, *Nestin*‐Cre and *Vil*‐Cre mice (Table 1), we found that the *Vil*‐Cre;*Pkd2* ^f3/f3^ mice developed disease phenotypes that were similar to human ADPKD (Fig. 1). Cre‐recombinase expressed in the *Vil*‐Cre transgenic mouse under the control of the mouse *villin‐*1 promoter is turned on at E12.5, when the kidney is starting to develop 30. Compared to control mice (*i.e*. *Pkd2* ^f3/f3^ mice without *Vil*‐Cre transgene) (Fig. 1A–C), *Pkd2* ^f3/f3^ mice with the *Vil*‐Cre transgene (*Vil*‐Cre;*Pkd2* ^f3/f3^) developed severe gross cysts in the kidneys, liver and pancreas (Fig. 1D–F). Anatomic and histological analyses of the *Vil*‐Cre;*Pkd2* ^f3/f3^ kidney (Fig. 1A *versus* D and G *versus* H) showed that renal failure was the likely cause of death in these mice, most of which died between 4 and 6 months of age (Fig. 1I). As this mouse model mimics the disease phenotype of human ADPKD patients and has an ideally temporal cystic phenotype, *Vil*‐Cre;*Pkd2* ^f3/f3^ mice can be used as an ADPKD model to study the pathogenesis of *Pkd2*‐associated ADPKD and to assess therapeutic responses.

### Characterization of cystic phenotypes in the *Vil*‐Cre;*Pkd2* ^f3/f3^ mice, an orthologous mouse model of ADPKD

**Figure 2:**
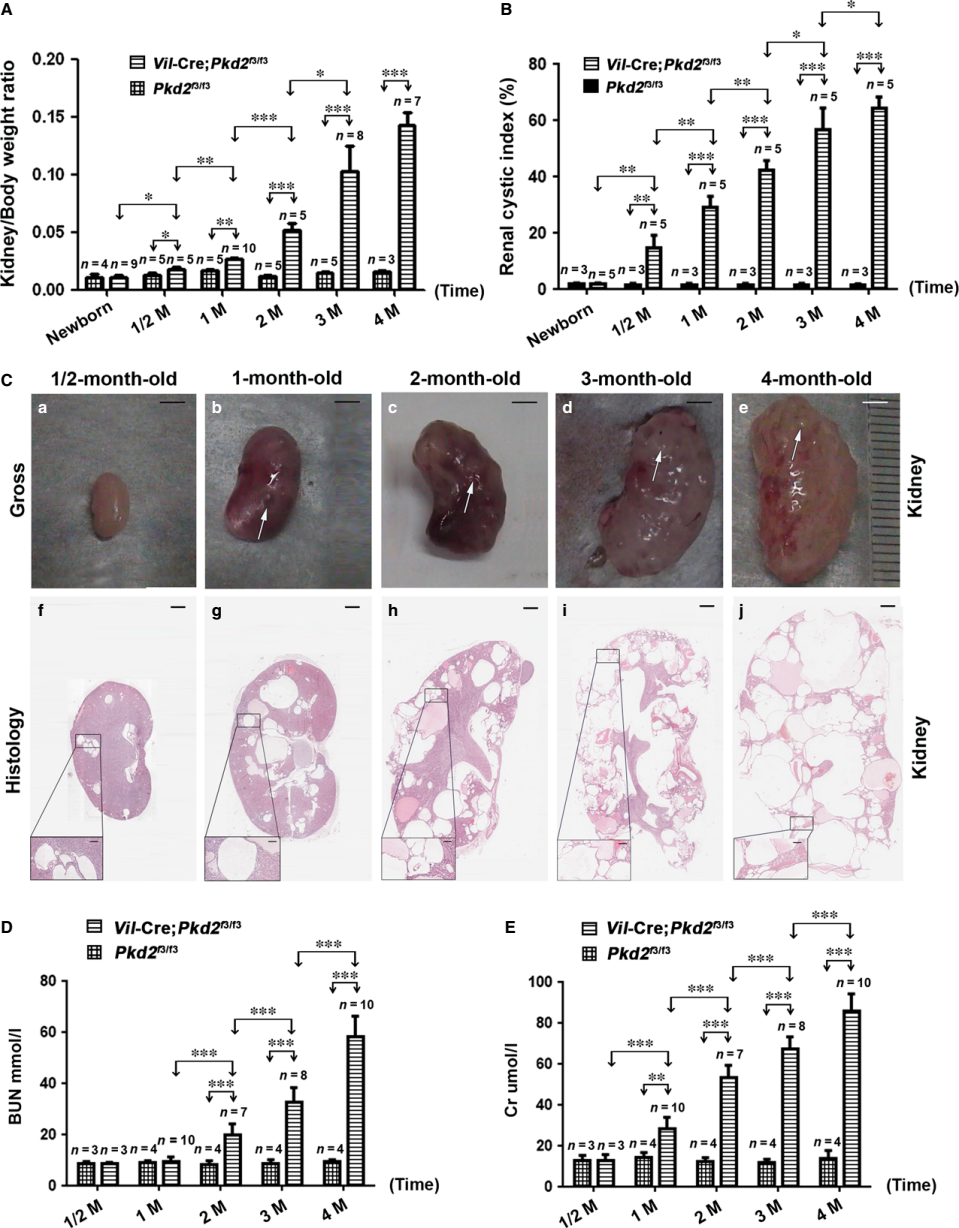
Characterization of the *Vil*‐Cre;*Pkd2* ^f3/f3^ kidney. (**A**) The kidney/body weight ratio for *Vil*‐Cre;*Pkd2* ^f3/f3^ newborns and for mice at ½, 1, 2, 3 and 4 months of age, compared to that of age‐matched control mice (*Pkd2* ^f3/f3^). (**B**) The renal cystic index of *Pkd2* ^f3/f3^ mice with or without the *Vil*‐Cre transgene allele at the ages shown. (**C**) The *Vil*‐Cre;*Pkd2* ^f3/f3^ kidney showed gross cysts (arrows) at 1 month of age (b) and massive gross cysts at 2–4 months (c‐e); histological results (f‐j) of the same kidneys were consistent with the gross examination. (**D**) BUN levels of *Vil*‐Cre;*Pkd2* ^f3/f3^ mice at ½, 1, 2, 3 and 4 months of age: BUN values rose significantly above those in control (*Pkd2* ^f3/f3^) mice at 2 months of age and continued to worsen with age. (**E**) Cr levels showed a similar pattern, starting at 1 month of age. *P < 0.05; **P < 0.01; ***P < 0.001. Bar: 3 mm in **C**a‐e; 600 μm in **C**f‐j; 100 μm in boxed areas. M: months.

To validate *Vil*‐Cre;*Pkd2* ^f3/f3^ mice as a suitable model for ADPKD, we systematically characterized the kidney, liver and pancreas by the kidney or liver/body weight ratio, cystic index and hepatorenal functions. In the kidneys, compared to control mice (*i.e*. *Pkd2* ^f3/f3^), the kidney/body weight ratio and the cystic index were significantly increased in *Pkd2* ^f3/f3^ mice with the *Vil*‐Cre transgene (*Vil*‐Cre;*Pkd2* ^f3/f3^). These mice had a significantly larger kidney/body weight ratio and renal cystic index by the time they were a half‐month old (*P* < 0.05) (Fig. 2A and B), indicating that cysts were already forming in the diseased kidneys at this early age.

Anatomic and histological analysis revealed that the *Vil*‐Cre;*Pkd2* ^f3/f3^ kidneys exhibited gross and microscopic cysts (Fig. 2C), in correlation with the kidney/body weight ratio and cystic index. By 4 months of age, normal parenchyma was almost entirely lost in the *Vil*‐Cre;*Pkd2* ^f3/f3^ kidney (Fig. 2Ce and j), indicating that these mice could suffer renal failure at this stage. To test renal function, we measured the blood urea nitrogen (BUN) and creatinine (Cr) levels in *Pkd2* ^f3/f3^ mice with and without the *Vil*‐Cre transgene. Compared to their *Pkd2* ^f3/f3^ littermates, the *Vil*‐Cre;*Pkd2* ^f3/f3^ mice had significantly higher BUN level at 2 months of age, but Cr level at 1 month of age (Fig. 2D and E). These results indicated that Cr might be more sensitive than BUN for detecting renal function damage in this disease model. In addition, we also used the nephron‐specific markers to clarify the renal segment of origin of the cysts in the *Vil*‐Cre;*Pkd2* ^f3/f3^ mice. Immunofluorescence staining revealed that the cyst‐lining cells in 3‐month‐old *Vil*‐Cre;*Pkd2* ^f3/f3^ kidneys were positive for all renal segments (Fig. S1), indicating that the renal cysts derived from all nephronic segments of the kidney.

By examination of the cystic liver and pancreas in *Vil‐*Cre;*Pkd2* ^f3/f3^ mice, we found no cysts in the liver of *Vil‐*Cre;*Pkd2* ^f3/f3^ mice before 1 month of age (Fig. S2Aa), or in the pancreas before 3 months of age (Fig. S2Ba‐c). However, gross cysts were observed in the liver starting at 2 months (Fig. S2Ab‐d) and in the pancreas starting at 4 months of age (Fig. S2Bd). Histological analyses revealed similar findings (Fig. S2Af‐h, Bh), but the cystic liver worsened rapidly with age. As *Vil‐*Cre;*Pkd2* ^f3/f3^ mice developed severe cysts in the liver, we also examined the liver/body weight ratio and measured the liver function using alanine aminotransferase (ALT). The liver/body weight ratio and liver function did not differ significantly between *Pkd2* ^f3/f3^ mice with or without the *Vil‐*Cre transgene (Fig. S2C‐D), indicating that as with human ADPKD patients, liver disease is not a life‐threatening factor in the mouse model. Our results showed that *Vil‐*Cre;*Pkd2* ^f3/f3^ mice not only exhibited a spatially and temporally appropriate cystic kidney phenotype for ADPKD, but also developed liver cysts as is often seen in human ADPKD patients.

### Effect of gender on disease severity in the *Vil*‐Cre;*Pkd2* ^f3/f3^ mice

Recent clinical studies have demonstrated that the progression of renal cysts in ADPKD may be influenced by gender 31, 32, 33, 34; the cystic disease appears to progress more quickly in males than in females. Male patients often develop end‐stage renal failure and require haemodialysis earlier than female patients, suggesting that the prognosis for women is better than that for men 4, 35, 36. To determine whether this gender difference was also present in the ADPKD mouse model, we analysed the Kaplan–Meier survival rate, kidney/body weight ratio and renal histology and function in male and female *Vil‐*Cre;*Pkd2* ^f3/f3^ mice. Notably, more than 50% of the female *Vil‐*Cre;*Pkd2* ^f3/f3^ mice survived to 5 months of age, approximately 1 month longer than their male counterparts (*P* < 0.01) (Fig. S3A), indicating a better survival rate and lifespan for females. In addition, histological analysis showed that the renal cystogenesis was more severe in the kidneys from male *Vil‐*Cre;*Pkd2* ^f3/f3^ mice (Fig. S3B). The renal cystic index and kidney/body weight ratio differed significantly between male and female *Vil‐*Cre;*Pkd2* ^f3/f3^ mice after 3 months of age (*P* < 0.05) (Fig. S3C‐D). Furthermore, the BUN and Cr levels in 4‐month‐old *Vil*‐Cre;*Pkd2* ^f3/f3^ males were significantly elevated over those in age‐matched females (*P* < 0.05) (Fig. S3E‐F). Thus, gender might influence cystogenesis in the ADPKD kidney, with female gender being a protective factor both in the animal model and in ADPKD patients 34, 37, 38.

### Validation of the *Vil*‐Cre;*Pkd2* ^f3/f3^ mice as an ADPKD animal model for drug treatment

**Figure 3:**
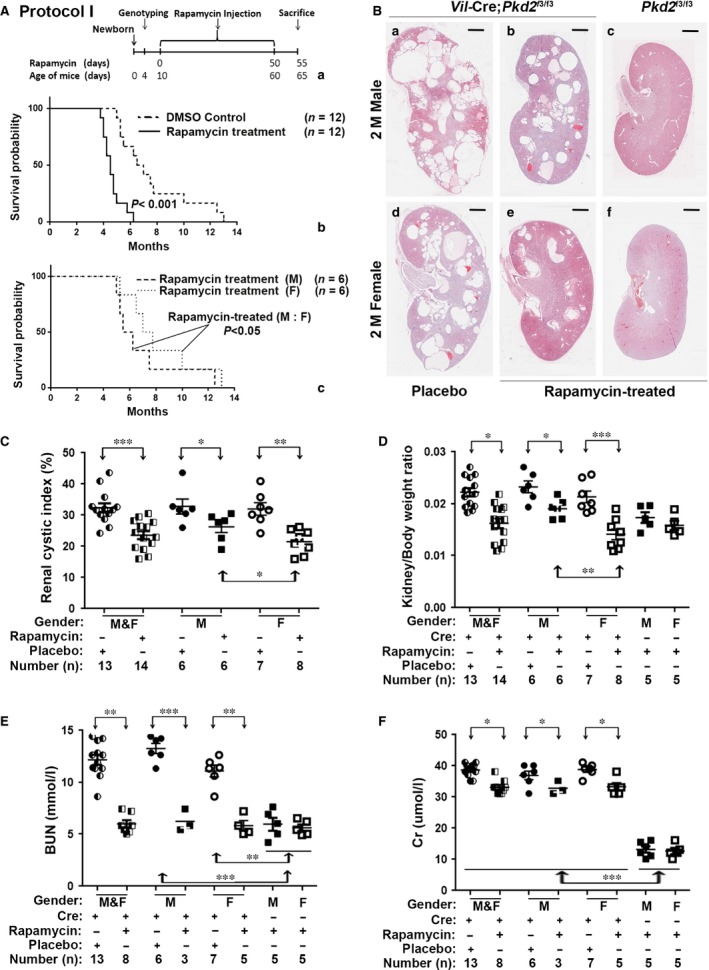
Rapamycin delayed cyst formation in *Vil*‐Cre;*Pkd2* ^f3/f3^ mice. (**A**) A diagram showing the initial treatment schedule for rapamycin injection (Protocol I) (a) in this study. Kaplan–Meier survival curves showed that compared to a placebo treatment, rapamycin significantly prolonged the lifespan in *Vil*‐Cre;*Pkd2* ^f3/f3^ mice (b) (*P* < 0.001), and that the lifespan of rapamycin‐treated *Vil*‐Cre;*Pkd2* ^f3/f3^ females was significantly longer than that of their male counterparts (c) (*P* < 0.05). (**B**) Histological examination of kidney sections from 2‐month‐old mice showed that cysts were reduced in the rapamycin‐treated compared to DMSO (placebo)‐treated *Vil*‐Cre;*Pkd2* ^f3/f3^ mice, and that kidneys from the rapamycin‐treated *Vil*‐Cre;*Pkd2* ^f3/f3^ females appeared less cystic than those of their male counterparts (b *versus* e). (**C**) The cystic index was significantly lower in rapamycin‐treated than placebo‐treated *Vil*‐Cre;*Pkd2* ^f3/f3^ mice (*P* < 0.001). Rapamycin produced a significantly stronger therapeutic response in the females than in the males (*P* < 0.05). (**D**) Kidney/body weight ratios for the mice in (**C**) were consistent with the cyst‐index findings. (**E**) BUN levels were significantly suppressed in the rapamycin‐treated compared to placebo‐treated *Vil*‐Cre;*Pkd2* ^f3/f3^ mice (*P* < 0.01). In both male and female *Vil*‐Cre;*Pkd2* ^f3/f3^ mice, rapamycin reduced the BUN to the level found in control (*Pkd2* ^f3/f3^) mice. (**F**) In the same mice, rapamycin significantly suppressed the Cr level (*P* < 0.05) but did not rescue Cr to the control level (*P* < 0.001). Bars: 600 μm in B. M: Male; F: Female.

Rapamycin is reported to significantly ameliorate the cystic phenotypes of diverse ADPKD animal models 10, 39, 40, 41, 42, 43, 44. To ensure that the ADPKD mouse model could be obtained therapeutic response, we treated *Vil*‐Cre;*Pkd2* ^f3/f3^ mice with rapamycin (50 mg/kg/day i.p.) (Protocol I, Fig. 3Aa). The Protocol I‐treated mice were given daily injections from postnatal day (P) 10 to 60; the control group was treated with the same injection and volume of DMSO solvent (placebo). The mice were killed 5 days (P65) after the end of treatment (ETR). Kaplan–Meier survival analysis showed that the *Vil*‐Cre;*Pkd2* ^f3/f3^ mice treated with rapamycin had a significantly longer lifespan than those treated with the placebo (*P* < 0.001) (Fig. 3Ab). As seen also in untreated mice (Fig. S3), the female rapamycin‐treated mice had a significantly higher survival rate than their male counterparts, with a median survival of about 7 months for females and 6 months for males (*P* < 0.05) (Fig. 3Ac).

Besides improving survival, intensive rapamycin treatment also significantly reduced the growth of renal cysts (Fig. 3B), the renal cystic index (Fig. 3C) and the kidney/body weight ratio (Fig. 3D) in *Vil*‐Cre;*Pkd2* ^f3/f3^ mice. The treatment response to rapamycin was significantly better in females (Fig. 3C and D), consistent with the survival analyses (Fig. 3Ac). We also investigated renal function and found that BUN and Cr levels were significantly improved in rapamycin‐treated as compared to placebo‐treated *Vil*‐Cre;*Pkd2* ^f3/f3^ mice (*P* < 0.01 and *P* < 0.05, respectively) (Fig. 3E and F). Improvements in BUN and Cr were similar in males and females. Interestingly, although rapamycin rescued the BUN level in *Vil*‐Cre;*Pkd2* ^f3/f3^ mice to that in control *Pkd2* ^f3/f3^ mice (Fig. 3E), rapamycin significantly reduced Cr (*P* < 0.05) but could not bring it down to the normal control levels (*P* < 0.001) (Fig. 3F). Thus, the intensive rapamycin protocol (Protocol I, Fig. 3Aa) could significantly improve the cystic phenotype of the *Vil*‐Cre;*Pkd2* ^f3/f3^ kidney, but might not fully rescue renal function.

### Enhanced rapamycin treatment improved the therapeutic response in our ADPKD animal model

**Figure 4:**
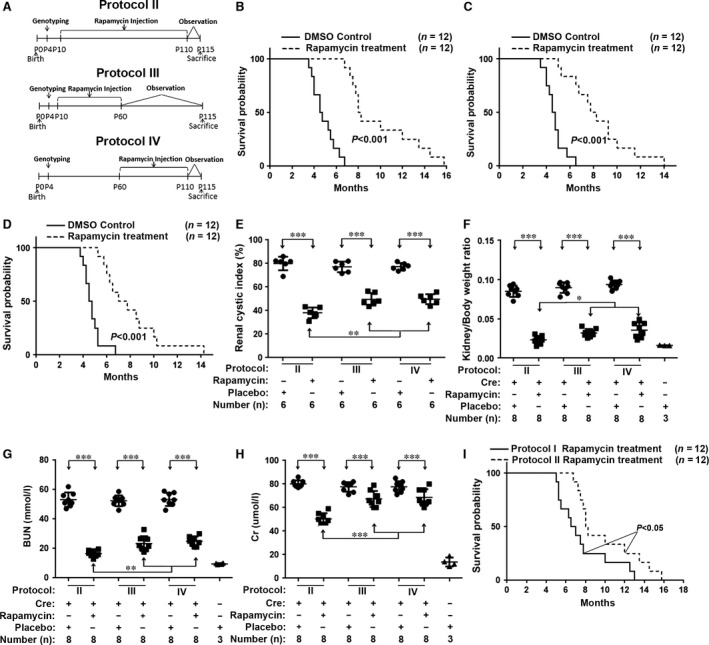
Increasing the dosage or duration of rapamycin treatment significantly improved the prognosis for the *Vil*‐Cre;*Pkd2* ^f3/f3^ mice. (**A**) Diagram showing the rapamycin treatment protocols (protocols II–IV) used in this study. (**B**) Kaplan–Meier survival curves showed that the lifespan was significantly prolonged in the *Vil*‐Cre;*Pkd2* ^f3/f3^ mice treated by Protocol II compared with those treated with DMSO (placebo) (*P* < 0.001); and that (**C**–**D**) survival was similarly improved for *Vil*‐Cre;*Pkd2* ^f3/f3^ mice treated with Protocol III *versus* IV (*P* < 0.001). (**E**) The cystic index for kidneys from *Vil*‐Cre;*Pkd2* ^f3/f3^ mice treated with Protocol II, III or IV. Protocol II provided the greatest improvement in the cystic index value (*P* < 0.01); (**F**) Kidney/body weight ratios for the same mice were consistent with the cystic index. Protocol II yielded the greatest improvement in the kidney/body weight ratio (*P* < 0.05). (**G**) In *Vil*‐Cre;*Pkd2* ^f3/f3^ mice, Protocol II significantly decreased the BUN level compared to that in DMSO (placebo)‐treated mice (*P* < 0.001). Protocol II decreased the BUN level significantly more than did Protocol III or IV (*P* < 0.01), returning the BUN nearly to the normal control level. (**H**) Similarly, Cr analysis of the same mice showed that Protocol II suppressed the abnormal Cr much more effectively than did Protocol III or IV (*P* < 0.001). (**I**) Kaplan–Meier survival curves for *Vil*‐Cre;*Pkd2* ^f3/f3^ mice treated with Protocol I or II, showing that Protocol II significantly prolonged the lifespan (*P* < 0.05).

Although preclinical studies have reported that rapamycin is an effective treatment for ADPKD in animal models 10, 40, 41, 42, 43, 44, 45, 46, 47, 48, 49, clinical trials of rapamycin treatment for ADPKD patients have been less successful 50, 51, 52, 53, 54, 55, 56, 57. These poor clinical results might be due to insufficient rapamycin 48, 55, 58. To verify this possibility in a cohort study *in vivo*, we treated *Vil*‐Cre;*Pkd2* ^f3/f3^ mice using three additional rapamycin protocols (Fig. 4A, protocols II–IV) that used the same rapamycin dose as in Protocol I (50 mg/kg/day i.p.) but varied the duration or timing of treatment. Protocol II extended the duration of treatment; mice received rapamycin injections from P10 to P110. In protocols III and IV, mice received rapamycin injections from P10 to P60 and from P60 to P110, respectively. Mice treated with protocols II–IV were killed at P115.

Kaplan–Meier survival analyses showed that the lifespans were significantly improved in *Vil*‐Cre;*Pkd2* ^f3/f3^ mice treated with protocols II–IV compared to those treated with the placebo (*P* < 0.001) (Fig. 4B–D). Rapamycin also significantly reduced the renal cyst growth (Fig. 4E) and kidney/body weight ratios (Fig. 4F) and improved BUN (Fig. 4G) and Cr values (Fig. 4H). Statistical analyses showed that Protocol II, with the longest duration of rapamycin treatment (100 days), yielded better results than did protocols III and IV, in which mice received rapamycin for the first or second 50 treatment days, respectively. Protocol II significantly reduced renal cyst growth (*P* < 0.01) (Fig. 4E), lowered the kidney/body weight ratios (*P* < 0.05) (Fig. 4F) and improved the BUN (*P* < 0.01) (Fig. 4G) and Cr (*P* < 0.001) (Fig. 4H) levels compared to protocols III and IV. We also compared the survival rates in the Protocol II group with those of other protocols (I, III and IV) (Figs 3Aa and 4A). Protocol II prolonged the lifespan in *Vil*‐Cre;*Pkd2* ^f3/f3^ mice to a median survival of about 8 months, compared to 6.5 months (*P* < 0.05) for Protocol I (Fig. 4I), and about 7 months for protocols III and IV (*P* < 0.001) (data not shown). Thus, extending the duration of rapamycin treatment significantly improved the prognosis of *Vil*‐Cre;*Pkd2* ^f3/f3^ mice.

### Rapamycin down‐regulates renal tubule proliferation in *Vil*‐Cre;*Pkd2* ^f3/f3^ mice by inhibiting cell‐cycle‐associated cyclins and cyclin‐dependent kinase

Previous studies demonstrated that rapamycin can effectively inhibit mTORC1 and its downstream factors 59, 60. Recent studies have shown that the mTOR pathway is dysregulated in ADPKD patients and in ADPKD animal models 45, 61. Rapamycin significantly inhibits cyst formation and greatly improves the prognosis for several ADPKD animal models 10, 40, 41, 42, 43, 44, 46, 47, 48, 49. However, the molecular link between polycystin dysfunction and mTOR signalling changes has not been clarified. In this study, we initially investigated the mTORC1 downstream indicators S6K1, S6rp, 4E‐BP1 and eIF4E in the *Vil*‐Cre;*Pkd2* ^f3/f3^ kidney with or without rapamycin treatment. Rapamycin significantly lowered the p‐S6K1 (T389), p‐S6rp (S235/236), p‐4E‐BP1 (S65) and p‐eIF4E (S209) levels in the *Vil*‐Cre;*Pkd2* ^f3/f3^ kidney (Fig. S4A‐H). However, Akt, a downstream marker of the mTORC2 complex in the mTOR pathway, did not appear to be affected (Fig. S4I‐J), indicating that rapamycin inhibited ADPKD cyst formation *via* the mTORC1 pathway, which might not be through mTORC2 40, 48.

The abnormal proliferation and apoptosis of cystic epithelial cells are hallmarks of ADPKD 62, 63, 64, 65. Consistent with previous studies 40, 41, 53, we found that although rapamycin did not affect apoptosis in the cyst‐lining epithelia of the *Vil*‐Cre;*Pkd2* ^f3/f3^ kidney (Fig. S5A and C‐D), rapamycin significantly suppressed the dramatic proliferation seen in the *Vil*‐Cre;*Pkd2* ^f3/f3^ renal epithelia (Fig. S5B and E‐F), indicating that rapamycin impeded cystic progression in the ADPKD model by inhibiting the proliferation of cyst‐lining epithelia.

To explore the molecular mechanism by which rapamycin suppresses epithelial proliferation, we have searched our previously established cDNA microarrays data between our E8 cell line (*Pkd2*‐null, *Pkd2* ^*−/−*^) and its maternally derived D3 cell line (*Pkd2* ^f*/−*^, *Pkd2* ^+*/−*^) 27. Of the signature genes, *Ccnd1* mRNA level encoding cyclin D1, whose function closely associates cell proliferation, showed a fourfold higher elevation in E8 than in D3 cells. To validate the microarray data, we performed quantitative PCR using mRNAs of E8 and D3 cells, as well as renal tissue mRNA from *Vil*‐Cre;*Pkd2* ^f3/3^ and its normal control *Pkd2* ^f3/f3^. We found that down‐regulating PC2 not only significantly increased *Ccnd1* expression, but also elevated all cell‐cycle‐associated cyclins and CDK1 (Fig. S5G‐H).

**Figure 5:**
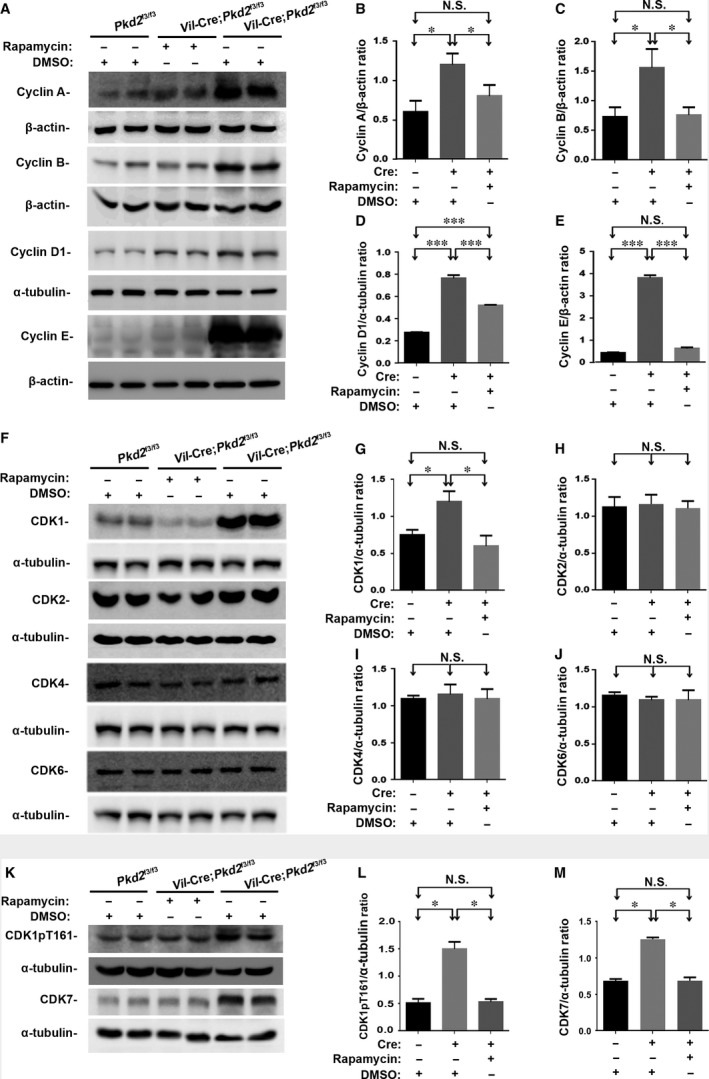
Rapamycin down‐regulates cell‐cycle‐associated cyclins and CDKs in *Vil*‐Cre;*Pkd2* ^f3/f3^ mice with or without Protocol II treatment. (**A**) Representative Western blots of kidney tissue lysates, showing that the aberrant elevation of cyclins A, B, D1 and E in *Vil*‐Cre;*Pkd2* ^f3/f3^ mice was suppressed by rapamycin treatment. (**B**–**E**) Normalized quantitative analyses using the densitometry values from the Western blots for cyclin A (**B**), cyclin B (**C**), cyclin D1 (**D**) and cyclin E (**E**). (**F**) Similar Western blots of the cell‐cycle‐associated CDK1, CDK2, CDK4 and CDK6 showed that only CDK1 was significantly elevated and suppressed by rapamycin treatment. (**G**–**J**) Normalized quantitative analyses using the densitometry values from the Western blots for CDK1 (**G**), CDK2 (**H**), CDK4 (**I**) and CDK6 (**J**). (**K**) Western blot analyses of the same tissues were performed with antibodies to phospho‐CDK1 (CDK1pT161), and its upstream factor CDK7. Both phospho‐CDK1 (CDK1pT161) and CDK7 were significantly elevated and suppressed by rapamycin treatment. (**L**–**M**) Normalized quantitative analysis using the densitometry values from the Western blots for CDK1pT161/α‐tubulin and CDK7/α‐tubulin. Statistical analysis indicated that compared to placebo treatment, rapamycin treatment significantly down‐regulated cyclins A, B, D1 and E and CDK1/CDK7 (including its activated form CDK1pT161) in the kidneys of *Vil*‐Cre;*Pkd2* ^f3/f3^ mice. N.S. = No significance; *P < 0.05; ***P < 0.001.

To confirm the finding from these mRNA assays was also affected in the mouse kidney tissue or in their epithelial cells with and without rapamycin treatment, we performed Western blot analyses of kidneys from control (*Pkd2* ^f3/f3^) mice and from rapamycin‐ or placebo‐treated *Vil*‐Cre;*Pkd2* ^f3/f3^ mice. The results revealed that all of the cell‐cycle‐associated cyclins (A, B, D1 and E) were abnormally up‐regulated in the *Vil*‐Cre;*Pkd2* ^f3/f3^ kidney tissue (Fig. 5A). These aberrantly up‐regulated cyclins were still further significantly suppressed by rapamycin to levels approaching those in the control kidney (Fig. 5B–E), indicating that rapamycin suppressed epithelial proliferation in the *Vil*‐Cre;*Pkd2* ^f3/f3^ kidney by inhibiting cell‐cycle‐associated cyclins. Interestingly, although rapamycin brought the cyclins A, B and E down almost to normal control levels (Fig. 5B,C and E), cyclin D1 was reduced to only approximately half of the excess level found in the untreated *Vil*‐Cre;*Pkd2* ^f3/f3^ kidney (Fig. 5D). The result suggested that rapamycin could not fully rescue all of the abnormally cell‐cycle‐associated cyclins in the *Vil*‐Cre;*Pkd2* ^f3/f3^ kidney.

Most cyclins couple with CDKs to form CDK/cyclin complexes which control kinase activity and substrate specificity 66, 67. CDK/cyclin complexes exert important role in driving cell cycle transition and regulating cell cycle progression. Our mRNA data showed that only CDK1, but not other cell‐cycle‐associated CDK2/4/6, was significantly affected (Fig. S5G‐H). We therefore tested all these CDKs in the same tested kidneys. Compared to the kidneys from control and rapamycin‐ or placebo‐treated *Vil*‐Cre;*Pkd2* ^f3/f3^ mice, the similar result to mRNA finding was obtained in the *Vil*‐Cre;*Pkd2* ^f3/f3^ kidney (Fig. 5F–J). This aberrantly up‐regulated CDK1 could also be significantly suppressed by rapamycin to level of the control kidney (Fig. 5G). As the tested CDKs’ function was usually activated by their phosphorylation sites, we therefore performed Western blot analyses to investigate all activated CDK forms, including CDK1pT161, CDK2pT160, CDK4pT172 and CDK6pY13. The same finding was seen in the tested renal tissues (Fig. 5K and L and Fig. S5I‐L). Because CDK‐activating kinase (CAK) phosphorylates CDK1pT161, we tested expressional level of a CAK catalytic subunit CDK7 in the same kidney. Similar results to CDK1pT161 were also seen in the tested renal tissues (Fig. 5K and M).

**Figure 6:**
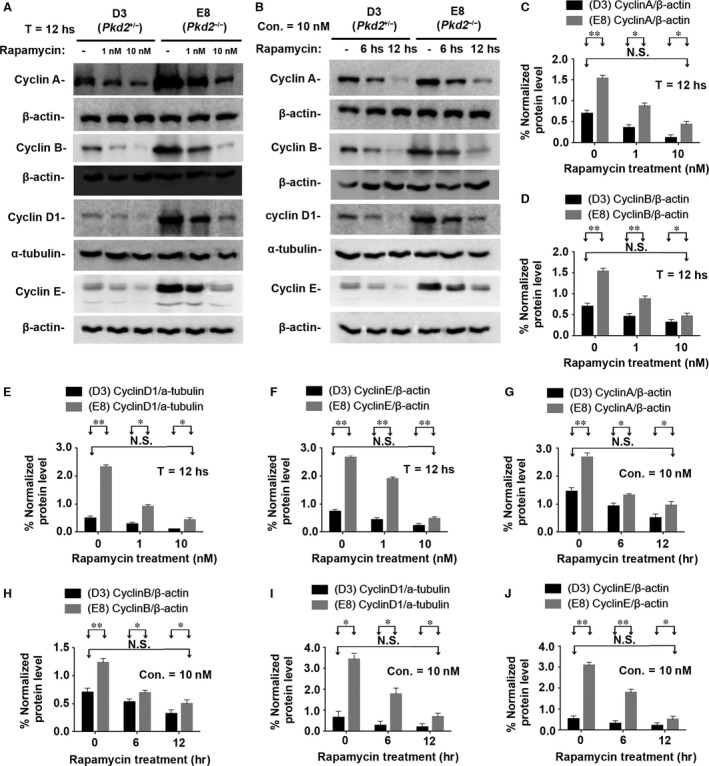
Rapamycin suppresses cell‐cycle‐associated cyclins *in vitro*. (**A**) Cell line E8 and its material derived cell line D3 were incubated with 0, 1 or 10 nM rapamycin for 12 hrs. Western blots of the lysates of D3 and E8 cells showed that rapamycin decreased the expression of cyclins A, B, D1 and E in a dose‐dependent manner. (**B**) D3 and E8 cells were incubated with 10 nM rapamycin for 0, 6 or 12 hrs. Western blots of the cell lysates showed that rapamycin significantly decreased the expression of cyclins A, B, D1 and E in a manner dependent on the duration of incubation. (**C**–**F**) Normalized quantitative analysis using the densitometry values from the Western blots in (**A**). (**G**–**J**) Normalized quantitative analysis using the densitometry values from the Western blots presented in (**B**). N.S. = No significance; *P < 0.05; **P < 0.01.

**Figure 7:**
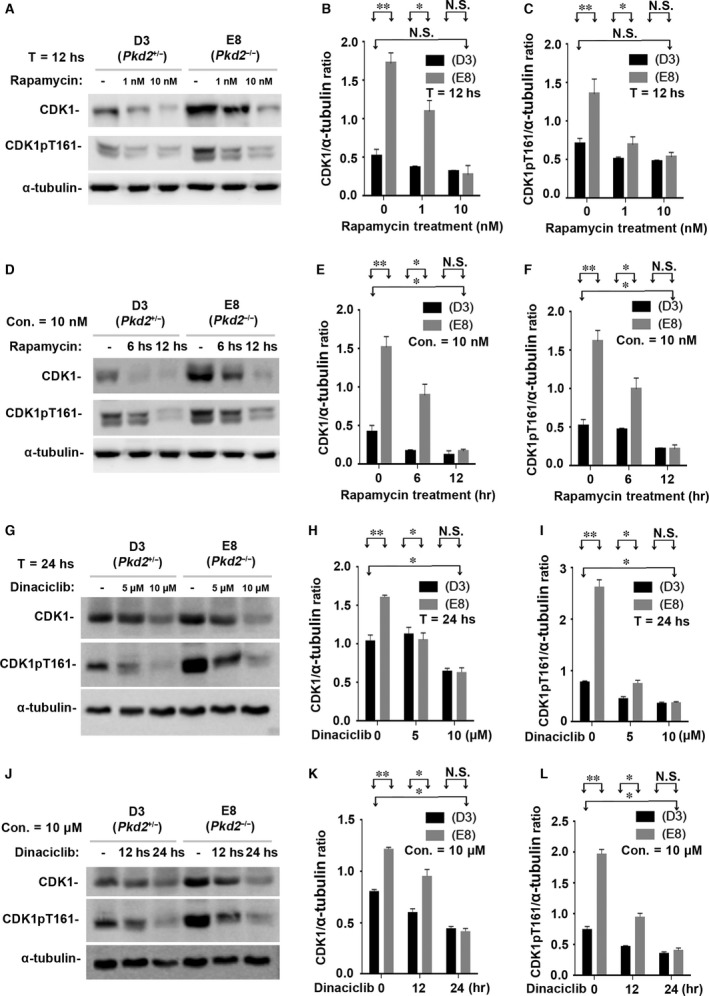
Rapamycin suppresses CDK1 and its activated form CDK1pT161 *in vitro*. (**A**) The same cell lines (E8 and D3) were also incubated with 0, 1, or 10 nM rapamycin for 12 hrs. Western blots of the lysates of E8 and D3 cells showed that rapamycin decreased the expression of CDK1 and its activated form CDK1pT161 in a dose‐dependent manner. (**B**–**C**) Normalized quantitative analysis using the densitometry values from the Western blots in (**A**). (**D**) The cell lines were also incubated with 10 nM rapamycin for 0, 6 or 12 hrs. Western blots of the cell lysates showed that rapamycin significantly decreased the expression of CDK1 and its activated forms CDK1pT161 in a manner dependent on the duration of incubation. (**E**–**F**) Normalized quantitative analysis using the densitometry values from the Western blots presented in (**D**). Other cell‐cycle‐associated CDKs (such as CDK2, CDK4 and CDK6) were not affected by PC2 expressional levels and rapamycin treatment (data not shown). (**G**) The same cell lines were cultured with CDK inhibitor Dinaciclib with 0, 5 and 10 μM for 24 hrs. Western blots of the cell lysates showed that Dinaciclib significantly inhibited the expression of CDK1 and its activated forms CDK1pT161 in a dose‐dependent manner. (**H**–**I**) Normalized quantitative analysis using the densitometry values from the Western blots presented in (**G**). (**J**) The cell lines were also cultured with CDK inhibitor Dinaciclib with 10 μM for 0, 12 or 24 hrs. Similar results to different concentrations were seen in Western blot assays on the different durations of Dinaciclib incubation. (**K**–**L**) Normalized quantitative analysis using the densitometry values from the Western blots presented in (**J**). N.S. = No significance; *P < 0.05; **P < 0.01.

To further validate this finding, we conducted cell‐based experiments to detect the cyclins (A, B, D1 and E) and CDKs (CDK1, CDK2, CDK4 and CDK6) in cells. We treated E8 (*Pkd2*‐null) and its maternally derived D3 (*Pkd2* ^+/*−*^) cells with rapamycin at different concentrations (non‐treated and treated with 1 nM or 10 nM for 12 hrs) (Fig. 6A and C–F and Fig. 7A–C) and durations (0, 6 and 12 hrs at 10 nM) (Fig. 6B and G–J and Fig. 7D–F). Increasing the rapamycin concentration appeared to produce a trend towards the delayed expression of the cell‐cycle‐associated cyclins (A, B, D1 and E) and CDK1/CDK1pT161 in both D3 and E8 cells. The highest rapamycin concentration (10 nM) brought all of these cyclins and CDK1/CDK1pT161 down to their normal control levels (Figs 6C–F and 7B‐C). Extending the duration of rapamycin treatment also increased its effect (Figs 6B and 7D). Treating *Pkd2*‐null cells (E8) with rapamycin at a concentration of 10 nM for the longest treatment duration (12 hrs) reduced the cyclins and CDK1/CDK1pT161 to the normal control levels (Figs 6G–J and 7E and F). In parallel, we also applied CDK1 inhibitor Dinaciclib to these cells with different concentrations and durations. The result showed that the highest concentration and the longest duration brought CDK1/CDK1pT161 down to their normal control levels (Fig. 7G–I and J–L). Thus, rapamycin significantly repressed the aberrant activation of cell‐cycle‐associated cyclins and CDK1/CDK1pT161 in *Pkd2*‐null cells in a dose‐ and time‐dependent manner.

## Discussion

We used a *Pkd2* conditional‐knockout model 27 to screen a spectrum of Cre transgenic mice driven by various kidney‐related promoters (Table 1) and identified the *Vil*‐Cre;*Pkd2* ^f3/f3^ ADPKD mouse model, which spatially and temporally mimics human ADPKD phenotypes. To validate *Vil*‐Cre;*Pkd2* ^f3/f3^ mice as a useful ADPKD model for preclinical trials, we used a rapamycin‐based treatment that is known to be effective for ADPKD mice 40, 41, 42. The response of the *Vil*‐Cre;*Pkd2* ^f3/f3^ mice to rapamycin was consistent with that reported for other ADPKD models, and the progression and timeline of the cystic phenotype and of renal failure in the *Vil*‐Cre;*Pkd2* ^f3/f3^ mice, along with the ease of evaluating the progression of the disease, make these mice a suitable model for evaluating and conducting preclinical trials of therapeutic interventions for ADPKD.

Rapamycin has been very effective for treating ADPKD mice, but has been less successful in clinical trials with ADPKD patients 26, 52, 53, 54, 57. Several explanations have been suggested for the different responses of mice and humans to rapamycin 41, 42, one being that to prevent adverse events, ADPKD patients have received lower doses (2–3 mg/kg) of rapamycin than those used in mouse models (5–10 or 100 mg/kg) 41, 42. Another possible reason for this discrepancy is that rapamycin has not been used as a primary treatment for humans in the early stages of ADPKD cyst development 52, 55, 68. To explore whether intensive rapamycin treatment could significantly improve disease phenotypes in an orthologous mouse model of ADPKD under similar conditions as those in human trials, we designed four rapamycin treatment protocols that varied the duration and timing of rapamycin treatment. Our results indicated that a longer duration of rapamycin treatment (Protocol II, from P10 to P110) provided the strongest therapeutic response than any of the other protocols (protocols I, III and IV, each with a duration of 50 days) in the ADPKD animal model. Protocol II yielded the largest increase in lifespan, significantly reduced the growth of renal cysts and significantly improved kidney function. Western blot analyses showed that rapamycin inhibited the activated forms of p‐S6K1, p‐S6rp, p‐4E‐BP1 and p‐eIF4E (indicators of mTORC1 activity; Fig S6) in a dose‐dependent manner, and this inhibition coincided with a significant improvement in the renal cystic phenotype in the rapamycin‐treated *Vil*‐Cre;*Pkd2* ^f3/f3^ mouse model. Thus, it is possible that an extended rapamycin treatment protocol would greatly benefit ADPKD patients with a *PKD2* mutation.

Interestingly, our results showed that early (P10‐60, Protocol I)‐ and late‐treatment (P60‐110, Protocol IV) protocols using the same rapamycin dosage provided similar improvements in survival, cystic phenotype and renal function. This finding indicated that starting rapamycin treatment during the early stages of cyst development might not be critical for a better prognosis in the ADPKD model. In addition, we also compared the effects of Protocol I with those of Protocol III, which used the same rapamycin dosage and treatment period as Protocol I (P10‐60) but includes a non‐treated observation period ending at P110. The responses to Protocol I and Protocol III did not differ significantly in the ADPKD model, indicating that rapamycin effect on cystogenesis of the animal model could be such sustained long after the last dose of rapamycin is administered.

Although rapamycin has shown clear benefits in preclinical experiments and clinical trials for ADPKD, the precise molecular mechanism by which PC2 regulates cell growth *via* mTOR and how rapamycin inhibits cyst growth and epithelial‐cell proliferation have not been clarified. To investigate the molecular mechanism by which rapamycin prevents proliferation, we examined the serial factors most likely to be involved in cell proliferation. Based on our microarray data and qPCR assay, we identified cyclin D1 was significantly up‐regulated. Subsequently, we also examined other cyclin‐family members and their cofactors CDKs, by which cell cycling was driven. We found that all of the cell‐cycle‐associated cyclins (A, B, D1 and E), activated CDK1 (CDK1pT161) and CDK7 (which involves in the phosphorylation of CDK1pT161) were significantly up‐regulated in *Pkd2*‐null renal cells and in the tissue of our ADPKD model mice and that these cyclins and CDK7/CDK1 were down‐regulated by rapamycin treatment. This finding indicated that the increased epithelial proliferation observed in the ADPKD kidney might result from abnormal activation of the mTORC1 pathway and the subsequent up‐regulation of cell‐cycle‐associated cyclins and activated CDK1.

**Figure 8:**
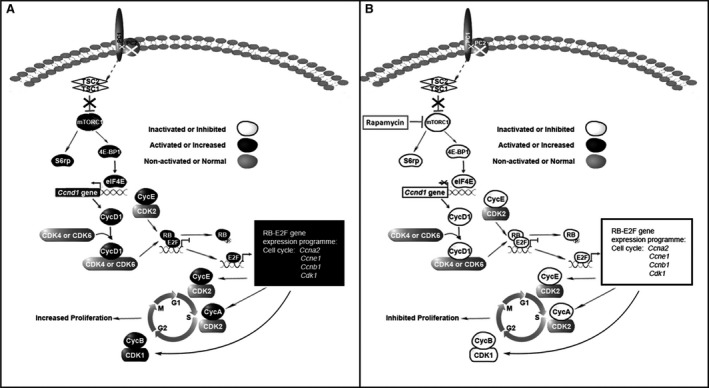
The molecular mechanism of the mTOR pathway in *Vil*‐Cre;*Pkd2* ^f3/f3^ mice, with or without rapamycin treatment. Based on our study and other group finding, we deduced the molecular link between mTOR signalling changes and the polycystin‐2 dysfunction in cystogenesis. (**A**) The loss of PC2 impairs normal PC1 function, which may in turn disrupt PC1's inhibitory effect on the mTORC1 pathway by disturbing interactions between PC1 and the tuberous sclerosis 2 gene product (TSC2). Activated mTORC1 promotes the phosphorylation of 4E‐BP1 and eIF4E, which activates cyclin D1 gene expression. Cyclin D1 couples with CDK4/6 and phosphorylates retinoblastoma protein (RB), thereby releasing (E2F), which promotes the expression of the other cell‐cycle‐associated cyclins (A, B and E) and CDK1, eventually leading to increased cell proliferation. (**B**) Rapamycin treatment suppressed all of the mTORC1 downstream factors, including cyclins D1, A, B, E and CDK1, thereby arresting aberrant cell proliferation.

Taking our findings together with those of other distinguished reports, we deduced the molecular mechanism of rapamycin effect on ADPKD as follows: PC2 dysfunction causes PC1 C‐terminal region (Cterm) to mislocalize and significantly down‐regulate PC1 expression (Fig. S7) 69, 70, 71. The down‐regulation of PC1 disrupts interactions between PC1 and tuberous sclerosis type 2 (TSC2) 45, 72, 73, 74; this abolishes the ability of the TSC complex to inhibit the mTORC1 pathway and thereby activates the mTORC1 downstream factors S6K1, S6rp, 4E‐BP1 and eIF4E (Figs S4 and S6) 75, 76. Activated eIF4E binds to the translation initiation site of the cyclin D1 gene, causing it to overexpress cyclin D1, a key regulator of cell growth (Figs 5D and 6E and I) 77, 78, 79. Cyclin D1 forms a complex with CDK4 or CDK6 to further phosphorylate the retinoblastoma protein (RB) and release E2F from the RB/E2F‐gene‐suppressing domain 80 (Fig. S8). E2F is a transcriptional factor that can initiate and enhance the expression of all of the other cell‐cycle‐associated cyclins, cyclin E (Figs 5E and 6F and J), cyclin A (Figs 5B and 6C and G) and cyclin B (Figs 5C and 6D and H) 66, 67, 81, 82, 83. These activated cyclins cooperate with their corresponding CDKs to promote the renal epithelial proliferation and induce cystogenesis in the ADPKD model (Fig. 8A). When rapamycin was applied, the PC2 inactivation‐induced overactivation of mTORC1 can be inhibited, and subsequently suppress all downstream factors of the mTORC1 pathway (Fig. 8B), thereby finally arresting abnormal cell proliferation in the orthologous mouse model of ADPKD.

In the light of our previous studies 27, 28, we identified and established a new ADPKD mouse model bearing *Pkd2* mutant alleles (*Vil*‐Cre;*Pkd2* ^f3/f3^). We systematically characterized the *Vil*‐Cre;*Pkd2* ^f3/f3^ mouse and verified its suitability as a model that mimics the phenotype and progression of human ADPKD. Using this new mouse system, we demonstrated that these animals responded to rapamycin in a time‐ and dose‐dependent manner: increasing the dosage or duration of treatment improved the therapeutic effect. We also demonstrated the molecular mechanism by which the mTOR inhibitor rapamycin arrests the aberrant epithelial proliferation in the ADPKD kidney, which resulted *de novo* from PC2 dysfunction. This newly developed ADPKD model will promote translational medicine from *in vivo* preclinical trials to ADPKD therapies and will also provide a platform for identifying new molecular targets for treating ADPKD based on the newly defined mTORC1–CDK1/cyclin axis.

## Conflict of interest

All the authors disclose no conflict.

## Author contributions

GW conceived and designed the experiments. AL, JM, XS and JM performed the experiments. AL, YX, LZ, XZ, DW, GM, CL and GW analysed and interpreted the data. AL, CL and GW wrote the manuscript.

## Supporting information


**Figure S1** Segmental origin of tubular cysts in the *Vil*‐Cre;*Pkd2* ^f3/f3^ kidney

**Figure S2** Extrarenal cystic phenotypes in *Vil*‐Cre;*Pkd2* ^f3/f3^ mice

**Figure S3** Gender affects disease severity in *Vil*‐Cre;*Pkd2* ^f3/f3^ mice

**Figure S4** Western blot analyses for mTOR downstream factors in the kidneys of 4‐month‐old *Vil*‐Cre;*Pkd2* ^f3/f3^ mice with or without Protocol II treatment

**Figure S5** Rapamycin decreases proliferation in renal cells in *Vil*‐Cre;*Pkd2* ^f3/f3^ mice. Apoptosis and proliferation in kidneys from DMSO‐treated and rapamycin‐treated (Protocol II) 4‐month‐old *Vil*‐Cre;*Pkd2* ^f3/f3^ mice were analysed by IF staining

**Figure S6** Rapamycin suppresses mTORC1 downstream indictors: phospho‐S6K1 (S6K1pT389), phospho‐S6rp (S6rppS235/236), phospho‐4E‐BP1 (4E‐BP1pS65) and phospho‐eIF4E (eIF4EpS209) in a dose‐dependent manner

**Figure S7** Lacking of PC2 down‐regulates PC1 expression *in vivo* and *in vitro*

**Figure S8** Rapamycin suppresses the up‐regulated RB/E2F pathway in the kidneys of 4‐month‐old *Vil*‐Cre;*Pkd2* ^f3/f3^ mice with or without Protocol II treatment

**Table S1** Genotyping and survival analyses of γGt‐, *Nestin*‐ and Ksp‐Cre;*Pkd2* ^f3/f3^ mice

**Appendix S1** Supplementary Method A‐D
