# Supplementary figures and images for: Rapamycin treatment dose‐dependently improves the cystic kidney in a new ADPKD mouse model via the mTORC1 and cell‐cycle‐associated CDK1/cyclin axis
Source: J Cell Mol Med. 2017 Feb 28;21(8):1619–35. doi: 10.1111/jcmm.13091 (PMC5543471; doi:10.1111/jcmm.13091)

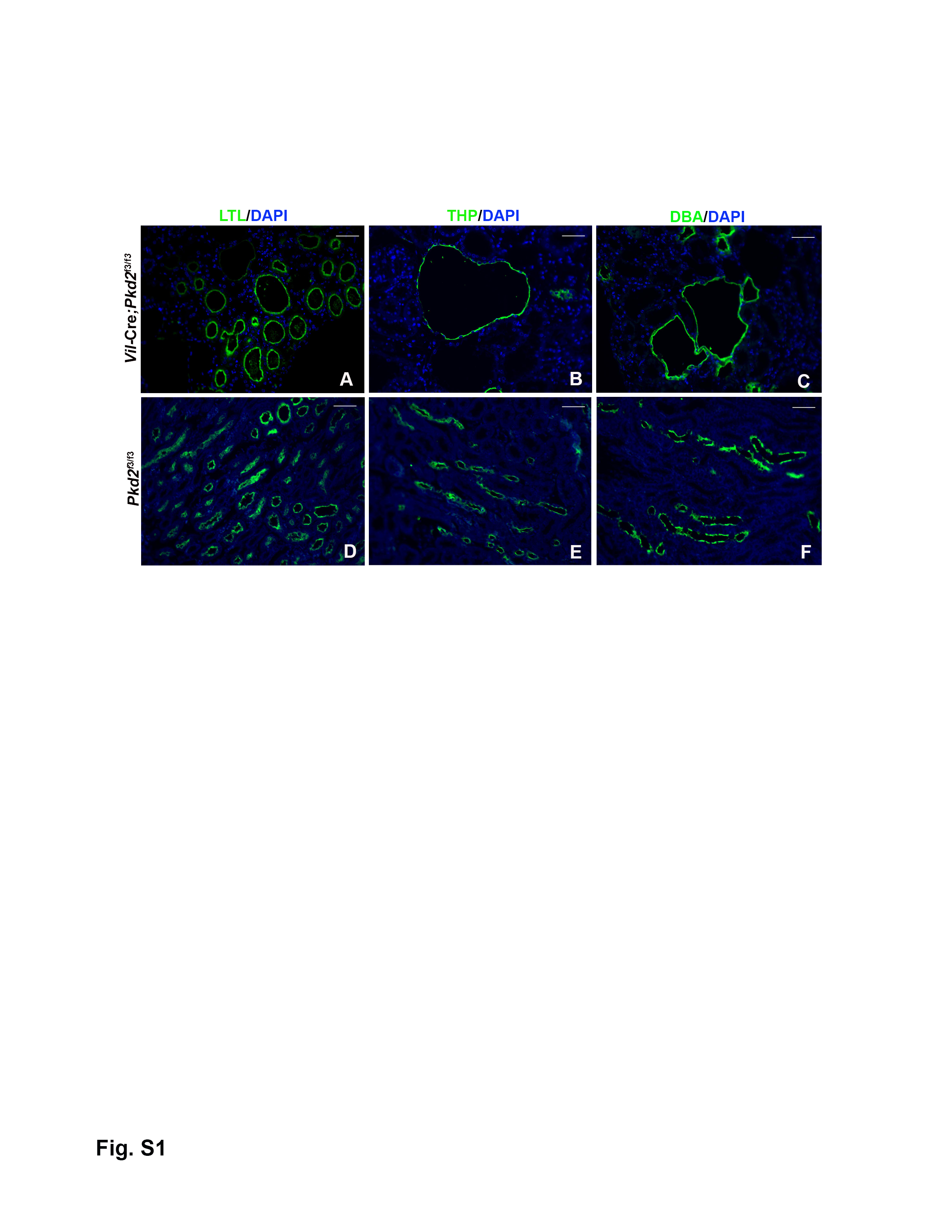

Supplement: Supplementary file 1 — Figure S1 Segmental origin of tubular cysts in the Vil‐Cre;Pkd2 f3/f3 kidney [file JCMM-21-1619-s001.tif]

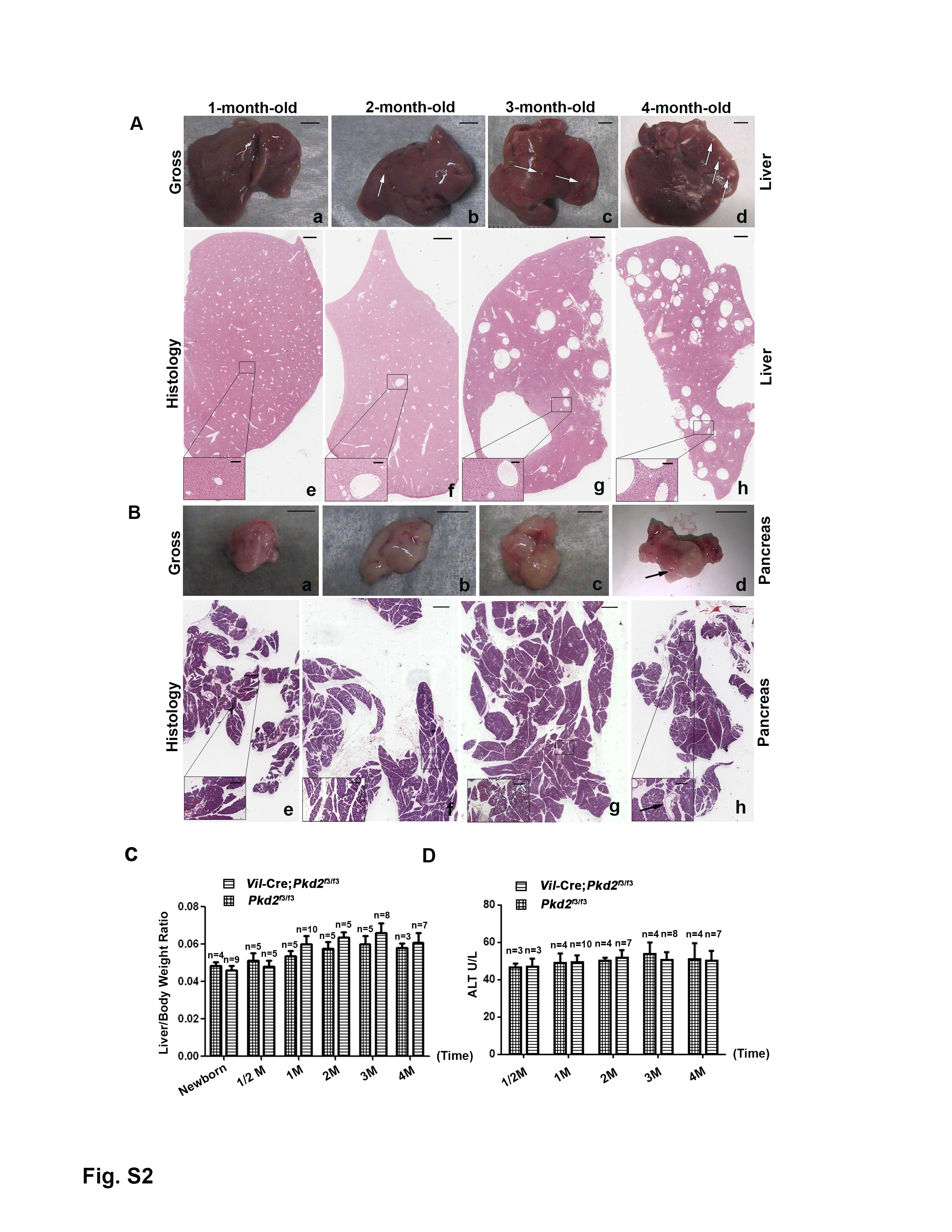

Supplement: Supplementary file 2 — Figure S2 Extrarenal cystic phenotypes in Vil‐Cre;Pkd2 f3/f3 mice [file JCMM-21-1619-s002.tif]

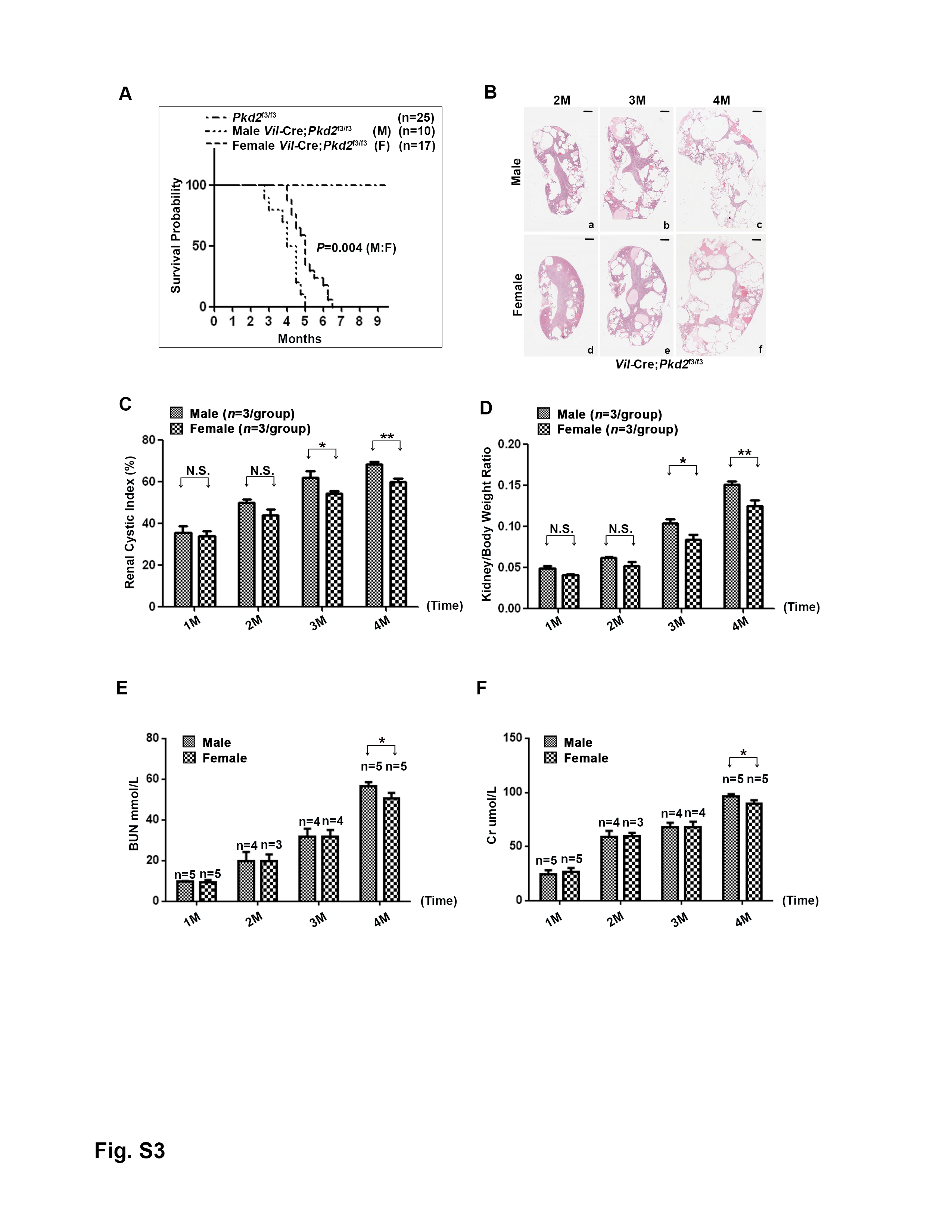

Supplement: Supplementary file 3 — Figure S3 Gender affects disease severity in Vil‐Cre;Pkd2 f3/f3 mice [file JCMM-21-1619-s003.tif]

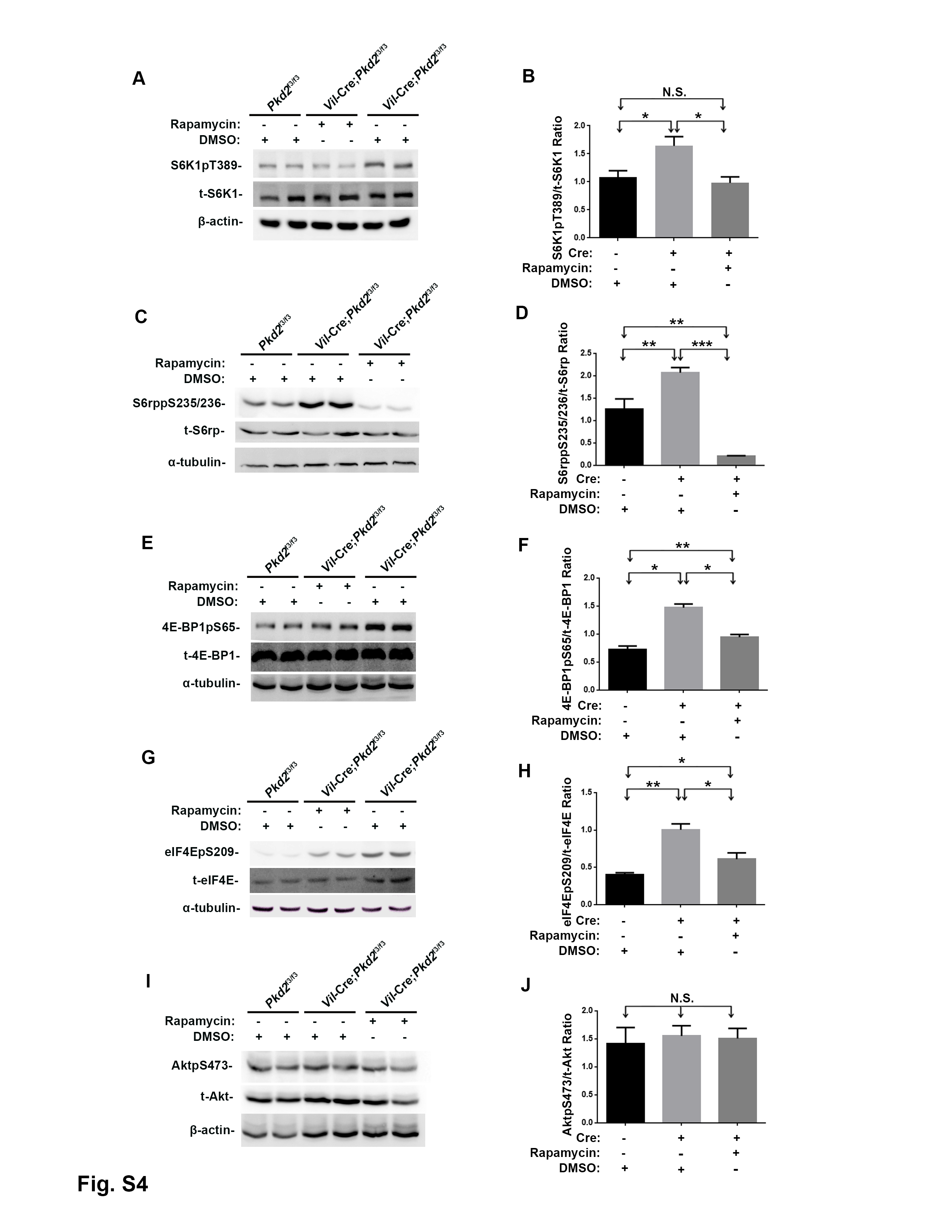

Supplement: Supplementary file 4 — Figure S4 Western blot analyses for mTOR downstream factors in the kidneys of 4‐month‐old Vil‐Cre;Pkd2 f3/f3 mice with or without Protocol II treatment [file JCMM-21-1619-s004.tif]

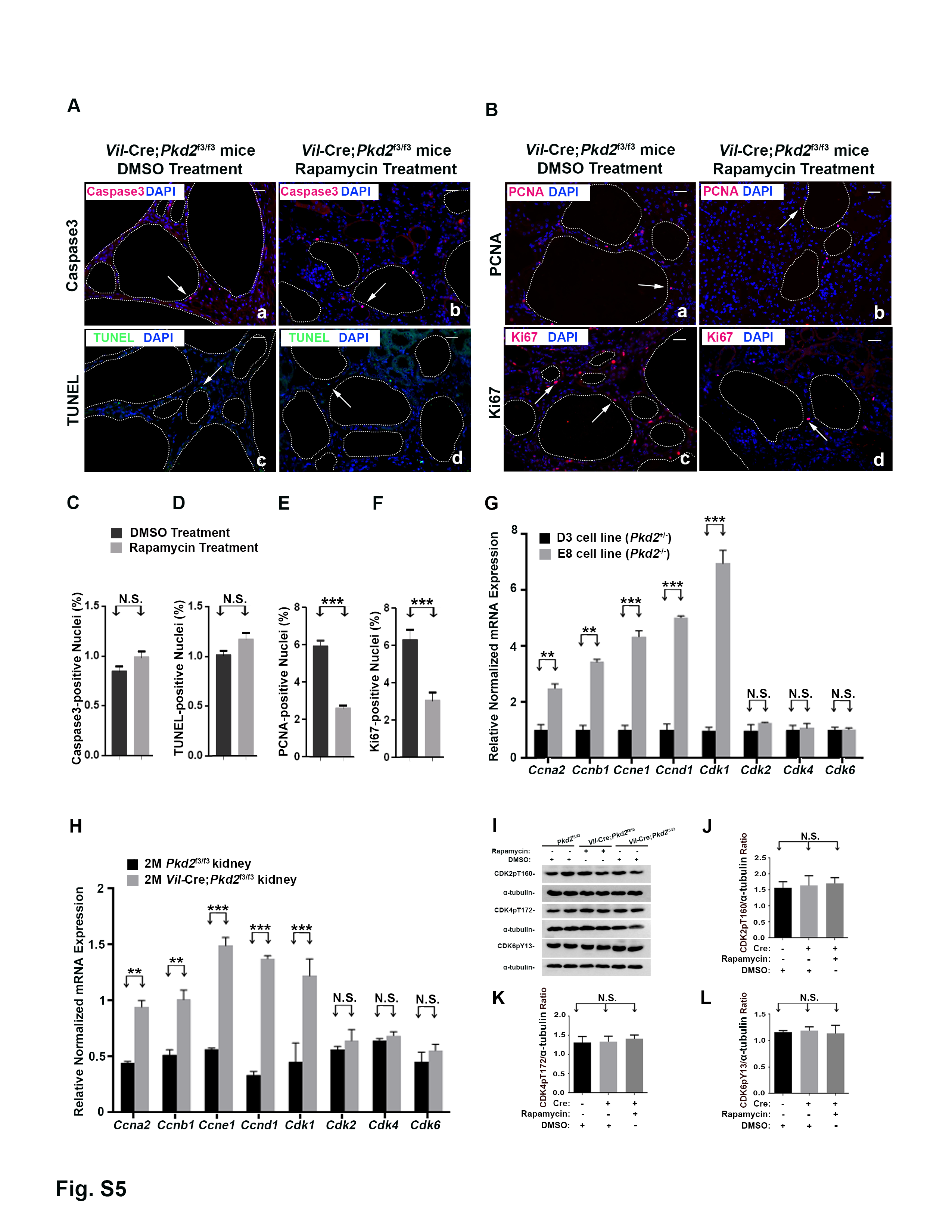

Supplement: Supplementary file 5 — Figure S5 Rapamycin decreases proliferation in renal cells in Vil‐Cre;Pkd2 f3/f3 mice. Apoptosis and proliferation in kidneys from DMSO‐treated and rapamycin‐treated (Protocol II) 4‐month‐old Vil‐Cre;Pkd2 f3/f3 mice were analysed by IF staining [file JCMM-21-1619-s005.tif]

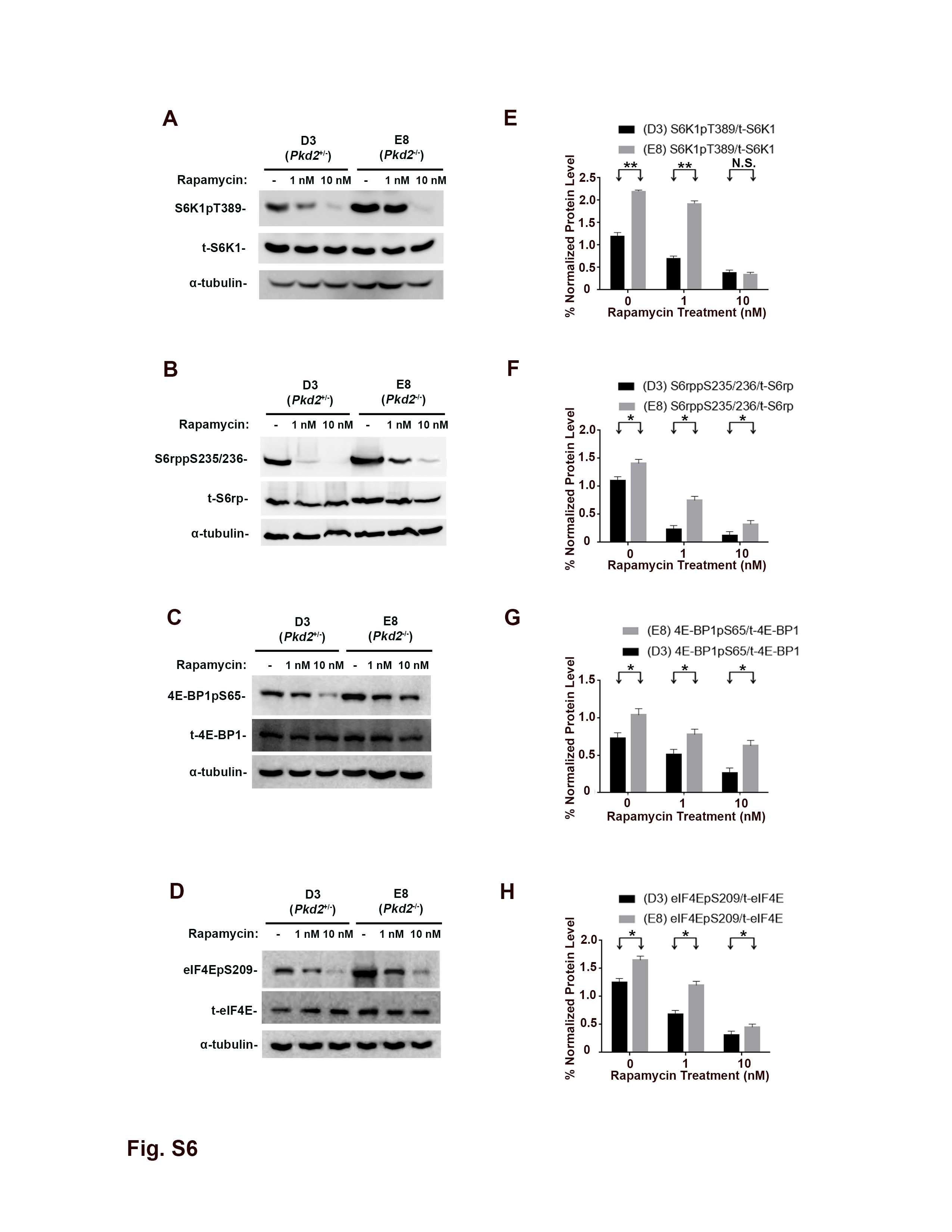

Supplement: Supplementary file 6 — Figure S6 Rapamycin suppresses mTORC1 downstream indictors: phospho‐S6K1 (S6K1pT389), phospho‐S6rp (S6rppS235/236), phospho‐4E‐BP1 (4E‐BP1pS65) and phospho‐eIF4E (eIF4EpS209) in a dose‐dependent manner [file JCMM-21-1619-s006.tif]

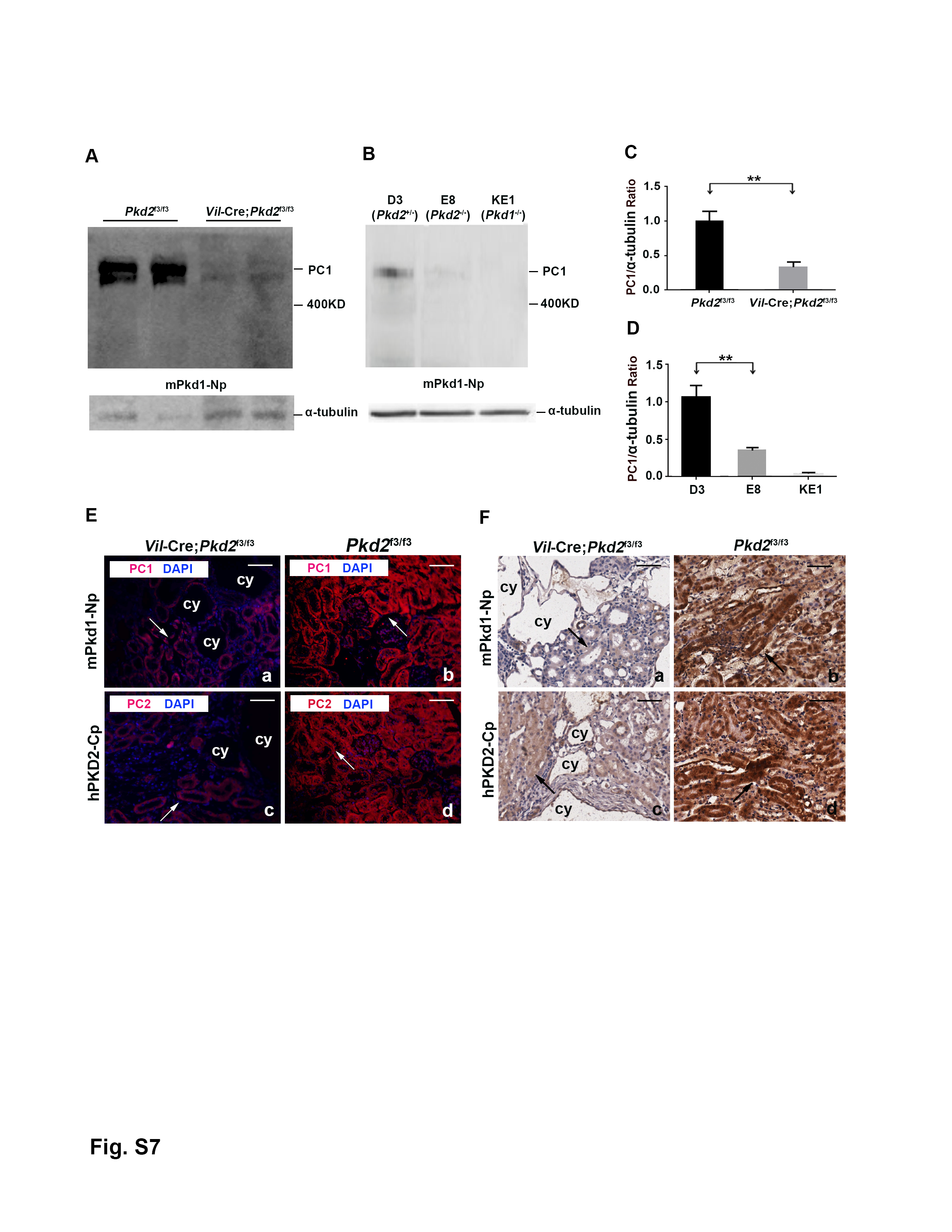

Supplement: Supplementary file 7 — Figure S7 Lacking of PC2 down‐regulates PC1 expression in vivo and in vitro [file JCMM-21-1619-s007.tif]

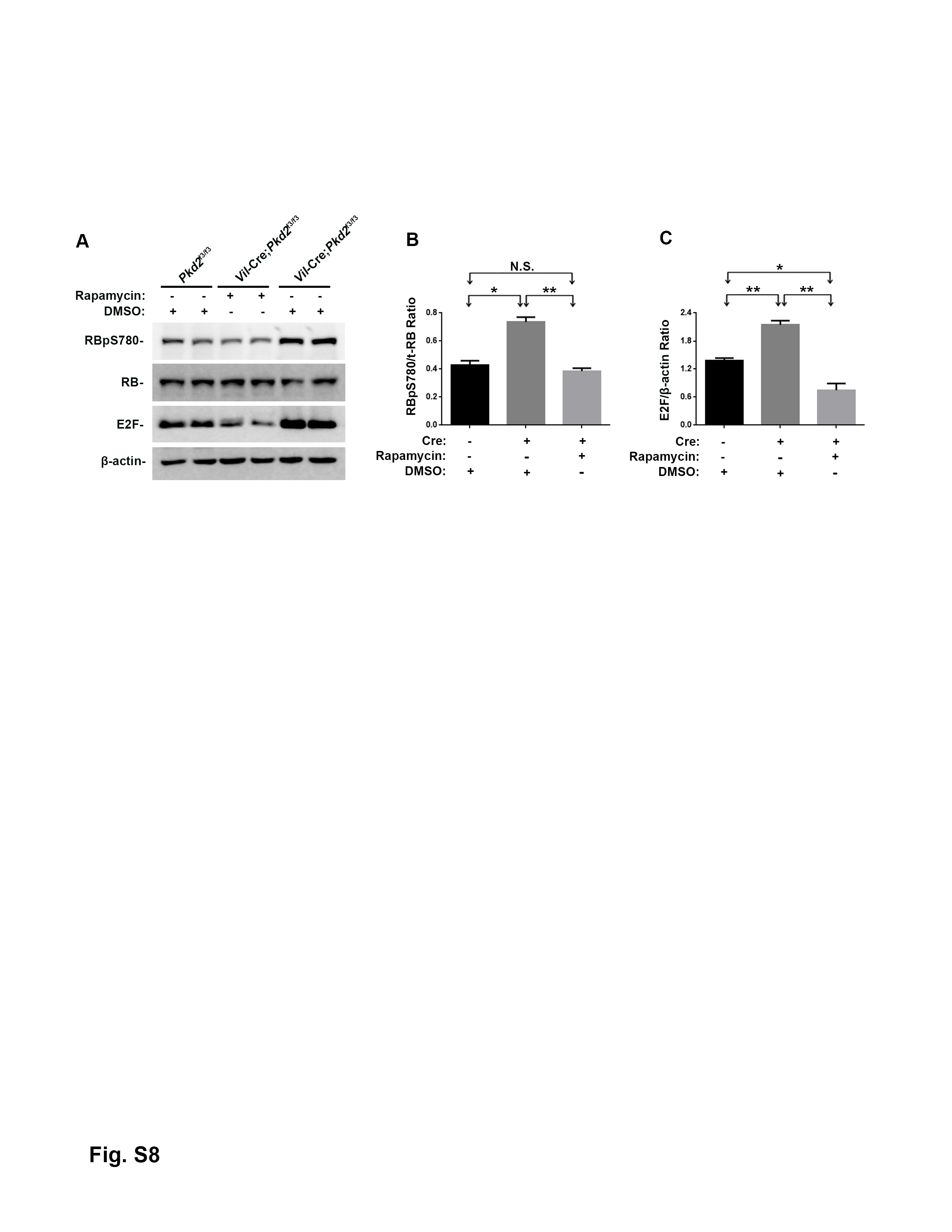

Supplement: Supplementary file 8 — Figure S8 Rapamycin suppresses the up‐regulated RB/E2F pathway in the kidneys of 4‐month‐old Vil‐Cre;Pkd2 f3/f3 mice with or without Protocol II treatment [file JCMM-21-1619-s008.tif]
