# Supplementary material for: Rapamycin treatment dose‐dependently improves the cystic kidney in a new ADPKD mouse model via the mTORC1 and cell‐cycle‐associated CDK1/cyclin axis
Source: J Cell Mol Med. 2017 Feb 28;21(8):1619–35. doi: 10.1111/jcmm.13091 (PMC5543471; doi:10.1111/jcmm.13091)
Supplement: Supplementary file 9 — Table S1 Genotyping and survival analyses of γGt‐, Nestin‐ and Ksp‐Cre;Pkd2 f3/f3 mice [file JCMM-21-1619-s009.docx]

Table S1. Genotyping and survival analyses of γGt-, *Nestin*- and Ksp-Cre;*Pkd2*^f3/f3^ mice.

| Type of Cre | Genotype | Age ≥4 weeks | Total |
| --- | --- | --- | --- |
| γGt-Cre; | **γGt-Cre;*Pkd2*^+/+^  n (%)** | 7 (29.2) | 24 |
|  | **γGt-Cre;*Pkd2*^+/f3^  n (%)** | 11 (45.8) |  |
|  | **γGt-Cre;*Pkd2*^f3/f3^  n (%)** | 6^a^ (25) |  |
| *Nestin*-Cre; | ***Nestin*-Cre;*Pkd2*^+/+^  n (%)** | 10 (23.2) | 43 |
|  | ***Nestin*-Cre;*Pkd2*^+/f3^ n (%)** | 22 (51.2) |  |
|  | ***Nestin*-Cre;*Pkd2*^f3/f3^  n (%)** | 11^b^ (25.6) |  |
| Ksp-Cre; | **Ksp-Cre;*Pkd2*^+/+^  n (%)** | 41 (39.4) | 104 |
|  | **Ksp-Cre;*Pkd2*^+/f3^  n (%)** | 53 (51) |  |
|  | **Ksp-Cre;*Pkd2*^f3/f3^  n (%)** | 10^c^ (9.6) |  |

^a^These mice were born at a frequency of 25% as the expected Mendelian ratio. The mice have not died until 12 month-old of age. n = number of mice

^b^These mice were also born at a frequency of approximately 25%. The mice likely died from renal failure at 9 to 15 month-old of age, their median survival time is 12 month-old. n = number of mice

^c^These mice were born at a frequency of approximately 10%, lower the expected Mendelian ratio, indicating a loss of 15% of the Ksp-Cre;*Pkd2*^f3/f3^ mice. These mice likely died from renal failure at birth to 5 month-old of age, their median survival time is 1.5 month-old. n = number of mice
