# Supplementary material for: Rapamycin treatment dose‐dependently improves the cystic kidney in a new ADPKD mouse model via the mTORC1 and cell‐cycle‐associated CDK1/cyclin axis
Source: J Cell Mol Med. 2017 Feb 28;21(8):1619–35. doi: 10.1111/jcmm.13091 (PMC5543471; doi:10.1111/jcmm.13091)
Supplement: Supplementary file 10 — Appendix S1 Supplementary Method A‐D [file JCMM-21-1619-s010.docx]

**Appendix S1**

**Supplementary Methods:**

**A. Mouse treatment and genotyping:** Male and female *Vil*-Cre;*Pkd2*^f3/f3^ mice were randomly divided into two groups, which were given vehicle DMSO (Sigma-Aldrich) or rapamycin (LC Laboratories) at 50 mg/kg/day i.p; four treatment protocols (I-IV) were used in the rapamycin-treated group (see Fig. 3Aa and Fig. 4A). All of the experiments were performed according to animal protocols approved by the Institutional Animal Care and Use Committee of Peking Union Medical College.

Mice with and without a *Pkd2* mutation were genotyped by PCR analysis of tail genomic DNA as previously described [27]. The primer pairs used for the *Pkd2*^f3^ allele were forward, 5’- TCT GAC TTG CAG ACT GTG GG -3’ and reverse, 5’-AGG TAG GGG AAG GTC AGG GTT GG -3’. The *Vil*-Cre transgene was genotyped by amplifying the PCR product with primers against the *villin*-1 promoter region (forward, 5’- GTGTGGGACAGAGAACAAACCG -3’ and reverse, 5’- TGCGAACCTCATCACTCGTTGC -3’).

**B. Material and reagents:**  Rabbit polyclonal antisera, which are against D682-V968 of human PC2 COOH-terminus (named hPKD2-Cp) and against E474-L640 of mouse PC1 NH_2_-terminus (named mPKD1-Np), were described in our previous studies [87,88]; β-actin antibody, anti-cyclin-A antibody, and DAPI (Sigma-Aldrich); anti-aquaporin-2 (AQP2), anti-total and phospho-S6 ribosomal protein (Serine 235/236) antibodies, anti-total and phospho-Akt (Serine 473) antibodies, anti-phospho-RB (Serine 780), anti-E2F, anti-cleaved caspase-3 (Asp 175) and anti-caspase-3 antibodies, anti-total and phospho-4E-BP1 (Serine 65) antibodies, anti-total and phospho-eIF4E (Serine 209) antibodies (Cell Signaling Technology); anti-total-RB, anti-CDK1, anti-CDK2, anti-CDK4, anti-CDK6, anti-cyclin B, anti-cyclin D1, and anti-cyclin E antibodies (Santa Cruz); anti-CDK7, anti-phospho-S6K1 (Thr 389), anti-S6K1, anti-phospho-CDK1 (Thr 161, anti-phospho-CDK2 (Thr 160), anti-phospho-CDK4 (Thr 172) and anti-phospho-CDK6 (Tyr 13) antibodies (ABclonal Technology); anti-Tamm-Horsfall glycoprotein (THP) antibody (Applied Biological Materials); fluorescein *Lotus tetragonolobuslectin* (LTL); *Dolichos Biflorus* agglutinin (DBA) (Vector Laboratories); anti-proliferating-cell nuclear antigen (PCNA) and anti-Ki67 antibodies (Abcam). Secondary antibodies included Cy2-conjugated donkey anti-sheep IgG and Cy2- and Cy3-conjugated goat anti-rabbit IgG (Jackson Laboratories); and peroxidase-conjugated rabbit anti-mouse IgG and peroxidase-conjugated goat anti-rabbit IgG (Promega). Dinaciclib (Selleck).

**C. Microarray analysis, qPCR assays and Western blot:** The isolated total RNA samples from E8 and D3 cell lines were then purified by RNeasy Mini Kit (QIAGEN), and 50 µg of the total RNA for each sample was sent to the Microarray Core facility at Vanderbilt University. In brief, 10 µg of labeled cRNA samples were hybridized onto an Affy mouse MOE430 array (45,101 different gene probes) (Affymetrix), using standard Microarray Core and Affymetrix protocols. Scanned raw data images were processed with GeneChip Operating Software (GCOS) 1.4. Probe set signal intensities were preprocessed and normalized by the Vanderbilt Microarray Shared Resource (VMSR) facility. An unsupervised hierarchical cluster with selected proliferation associated concordant genes was identified using TIGR MeV program (PMID: 12613259).

For qPCR, the total RNA was isolated from the tissues of transgenic and wild-type mice using Trizol (Invitrogen). The cDNA was then generated from the total RNA (2 μg) using SuperScript® II reverse transcriptase according to the manufacturer’s protocol (Invitrogen). The primer pairs used for analysis were: (forward) 5’-TGC AGC CTG CAA ACT GTA AGG-3’ and (reverse) 5’-GCA GCT CCA GCA ATG AGT GAA-3’ for Cyclin A2; (forward) 5’-ACT TCC TCC GTA GAG CAT C-3’ and (reverse) 5’-GCA GAG TTG GTG TCC ATT C-3’ for mouse Cyclin B1; (forward) 5’-TGT TTT TGC AAG ACC CAG ATG A-3’ and (reverse) 5’-GGC TGA CTG CTA TCC TCG CT-3’ for Cyclin E1; (forward) 5’-GCG TAC CCT GAC ACC AAT CTC-3’ and (reverse) 5’-CTC CTC TTC GCA CTT CTG CTC-3’ for Cyclin D1; (forward) 5’-ACT CCA GGC TGT ATC TCA TC-3’ and (reverse) 5’-CAA GTC TCT GTG AAG AAC TCG-3’ for CDK1; (forward) 5’-TCT GCT CTC ACG GGC ATT C-3’ and (reverse) 5’-AGC TGG AAC AGA TAG CTC TTG ATG A-3’ for CDK2; (forward) 5’-GCT GGA GGC CTT TGA ACA TC-3’ and (reverse) 5’-CCC GAT CAG TTC GGG AAG TAG-3’ for CKD4; (forward) 5’-GGA CTT TCT TCA TTC ACA CCG-3’ and (reverse) 5’-GAC CAC TGA GGT TAG GCC A-3’ for CDK6 and (forward) 5’- GAC CAC AGT CCA TGC CAT CAC -3’ and (reverse) 5’- TCC ACC ACC CTG TTG CTG TA -3’ for GAPDH as a mRNA loading control. The PCR products were separated by 1.5% agarose gel electrophoresis. Quantitative PCR was performed using the iCycler iQ Real-Time PCR Detection System with the iQ SYBR Green Supermix kit (Bio-Rad).

For western blot, fresh tissues or cultured cells were homogenized in RIPA buffer (Sigma-Aldrich), and protein concentrations were determined by BCA assay (Pierce). Samples were separated by SDS-PAGE and electro-transferred to a nylon membrane (Perkin Elmer). The membrane was incubated with primary antibodies at room temperature for 4 hrs and with peroxidase-conjugated secondary antibodies for 1 hrs. Antibodies were detected with enhanced chemiluminescence (ECL) (Pierce). Western blot results were quantified using the densitometry values of the immunoreactive bands for targeted proteins, normalized to the total β-actin or α-tubulin (loading control) or of the phosphorylated form of a protein versus its total protein amount.

**D. Statistics and detailed procedures for measuring cystic index, proliferation, apoptosis, BUN and Cr:** Five representative 200**×**HE images per mouse were captured from renal sections of the kidney of untreated or rapamycin-treated *Vil*-Cre;*Pkd2*^f3/f3^ mice and control *Pkd2*^f3/f3^ mice. The images were opened in Photoshop software, converted to grayscale, and resized to 800**×**598 pixels. A grid was placed over the image to create a total of 1064 individual points. The points bisecting cyst epithelium and non-cystic epithelium were counted, and the percentage of total points that bisected the cyst epithelium and non-cystic epithelium was calculated as the cystic index.

To examine cell proliferation, kidney samples were fixed in 4% buffered paraformaldehyde (Sigma-Aldrich), embedded in paraffin, and cut into sections on a cryostat. The sections were deparaffinized, rehydrated, and washed in PBS three times for 5 min. each. Epitopes were unmasked by microwave retrieval with citrate buffer (pH 6.0). The sections were then immersed in 3% hydrogen peroxide for 10 min. to inactivate endogenous peroxidase, washed with PBS three times for 5 min. each, and blocked with 2% BSA for 1 hr. The sections were then incubated with anti-PCNA and anti-Ki67 antibodies overnight at 4°C, then incubated with a Cy3-conjugated goat anti-rabbit secondary antibody and stained with DAPI to identify all the cell nuclei. Proliferation was measured by counting the PCNA- and Ki67-positive nuclei on the cyst-lining epithelia. Nuclei were counted in three randomly chosen high-power fields (20**×**) for each sample.

Apoptosis was detected using the DeadEnd™ Fluorometric TUNEL kit (Promega) and a cleaved caspase-3 antibody, according to the manufacturer's instructions. Kidney samples were placed on slides, deparaffinized and rehydrated, fixed in 4% formaldehyde in PBS for 15 min., and then permeabilized with Proteinase K for 10 min. After being washed in PBS, the samples were equilibrated and labeled for 60 min. The samples were then immersed in 2**×** SSC to stop the reaction, and counterstained with DAPI. Positive apoptosis signals were detected by fluorescence microscopy. Apoptotic cells were counted in the same manner as the proliferation markers.

For BUN and Cr measurements, in accordance with Institutional Animal Care and Use Committee regulations, all mice were anesthetized with isoflurane preceding cardiac puncture. Blood samples were centrifuged and serum was collected. BUN/Cr levels were analyzed by the clinical laboratory of Cancer Hospital, Chinese Academy of Medical Sciences.

For statistics, all assays were repeated at least three times in duplicate or triplicate. Graphic data are presented as the mean ± SD unless otherwise stated. Statistical analysis was performed where appropriate using the Student's *t-*test or one-way analysis of variance (ANOVA) followed by Tukey's multiple comparison test. Differences with *P*-values < 0.05 were considered statistically significant (N.S., no significance; *: *P* < 0.05; **: *P* < 0.01; ***: *P* < 0.001).

**Figure S1.** Segmental origin of tubular cysts in the *Vil*-Cre;*Pkd2*^f3/f3^ kidney. (**A**) The origins of cystic tubules in the *Vil*-Cre;*Pkd2*^f3/f3^ kidney were determined by immunofluorescence staining for the following nephronic markers: *Lotus tetragonolobus*lectin (LTL) for the proximal tubules, THP for the distal tubules, and *Dolichosbiflorus agglutinin* (DBA) for the collecting ducts. (**A**) LTL staining of a 3-month-old *Vil*-Cre;*Pkd2*^f3/f3^ kidney, showing dilated tubules surrounding the cyst that originated from proximal tubules. (**B**) THP labeling was positive in the same kidney. (**C**) DBA-positive cysts were also observed in the kidney. (**D**-**F**) Age-matched *Pkd2*^f3/f3^ mice kidneys were used as the control. Bars: 60 μm.

**Figure S2.** Extrarenal cystic phenotypes in *Vil*-Cre;*Pkd2*^f3/f3^ mice. (**A**) Cystic phenotypes in the *Vil*-Cre;*Pkd2*^f3/f3^ liver: there were no obvious gross cysts at (a) 1 month of age, but (b) sporadic gross cysts (arrow) were observed in the liver at 2 months of age. (c-d) At 3 and 4 months of age, multiple gross cysts (arrows) were found in the diseased liver. (e-h) Histological analysis and gross examination revealed similar findings: cysts were found in the 2-month-old liver (see the boxed area), and massive cysts (arrows) were present at 3 and 4 months of age. (**B**) Cystic phenotypes in the *Vil*-Cre;*Pkd2*^f3/f3^ pancreas. (a-c) No obvious gross cysts were seen in the *Vil*-Cre;*Pkd2*^f3/f3^ pancreas until 3 months of age. Sporadic gross cysts (arrow) were observed at 4 months of age (d). (e-h) Histological results were consistent with the gross examination. Insets in the lower-left corners show magnified views of the box in the main panels. *n*=5 for each age. (**C**-**D**) Liver/body weight ratios and ALT levels were not significantly different in *Pkd2*^f3/f3^ versus *Vil*-Cre;*Pkd2*^f3/f3^ mice at the ages shown. Bar: 3 mm in Aa-d and Ba-d; 600 μm in Ae-h and Be-h; 100 μm in boxed areas. M: months.

**Figure S3.** Gender affects disease severity in *Vil*-Cre;*Pkd2*^f3/f3^ mice. (**A**) Kaplan-Meier survival analysis of *Vil*-Cre;*Pkd2*^f3/f3^ mice by gender showed that females had a significantly higher survival rate (*P* < 0.01). (**B**) Histological examination of kidneys from male and female *Vil*-Cre;*Pkd2*^f3/f3^ mice at the ages shown; cyst progression was more rapid in males. (**C**) The renal cystic index was significantly different between male and female *Vil*-Cre;*Pkd2*^f3/f3^ mice 3 months of age and older (**P* < 0.05; ***P* < 0.01). (**D**) Kidney/body weight ratios of *Vil*-Cre;*Pkd2*^f3/f3^ mice were consistent with the cystic index. (**E**-**F**) Renal function analyses showed that at 4 months of age, the BUN and Cr levels of *Vil*-Cre;*Pkd2*^f3/f3^ males were significantly higher than those of their female counterparts (*P* < 0.05). Bars: 600 μm in **B**.

**Figure S4.** Western blot analyses for mTOR downstream factors in the kidneys of 4-month-old *Vil*-Cre;*Pkd2*^f3/f3^ mice with or without Protocol II treatment. Duplicate protein lysates from control (*Pkd2*^f3/f3^) kidneys and from rapamycin-treated and placebo-treated *Vil*-Cre;*Pkd2*^f3/f3^ kidneys were immunoblotted with antibodies against (**A**) phospho-S6K1 (S6K1pT389) and total S6K1 (t-S6K1), (**C**) phospho-S6rp (S6rppS235/236) and total S6rp (t-S6rp), (**E**) phospho-4E-BP1 (4E-BP1pS65) and total 4E-BP1 (t-4E-BP1), (**G**) phospho-eIF4E (eIF4EpS209) and total eIF4E (t-eIF4E), and (**I**) phospho-Akt (AktpS473) and total Akt (t-Akt); α-tubulin and β-actin was used as a protein loading control. (**B**, **D**, **F**, **H**, and **J**) Normalized quantitative analyses were performed using densitometry values from the western blots of **A**, **C**, **E**, **G**, and **I**, respectively.

**Figure S5.** Rapamycin decreases proliferation in renal cells in *Vil*-Cre;*Pkd2*^f3/f3^ mice. Apoptosis and proliferation in kidneys from DMSO-treated and rapamycin-treated (Protocol II) 4-month-old *Vil*-Cre;*Pkd2*^f3/f3^ mice were analyzed by IF staining. (A) Apoptosis (arrows) was detected by (a, b) staining with an anti-cleaved caspase-3 antibody and (c, d) by TUNEL assay. (**B**) In the same mice, proliferation (arrows) was assessed by staining for (a, b) PCNA and (c, d) Ki67. (**C**-**D**) Stained cyst-lining epithelial cells were counted in 8 different 20**×** microscopic fields. The rate of apoptosis did not differ significantly between the placebo-treated and rapamycin-treated *Vil*-Cre;*Pkd2*^f3/f3^ kidney. (**E**-**F**) Proliferation rates were assessed by counting stained cells as in **C**. Proliferation was significantly reduced in the rapamycin group compared to the placebo-treated group (*P* < 0.001). *n*=8/genotype. (**G**) Quantitative PCR (qPCR) of mRNAs extracted from the E8/D3 cell lines, using all cell-cycle-associated cyclins’ and CDKs’ primers. The results showed that all cell-cycle-associated cyclins and CDK1 were significantly increased in E8 (*Pkd2*-null) cell line, but other CDK (including CDK2, 4 and 6) mRNA expression levels were not affected by loss of PC2. (**H**) Compared to control mice (*Pkd2*^f3/f3^), qPCR also showed a similar finding in the 2-month-old *Vil*-Cre;*Pkd2*^f3/f3^ kidney tissue. (**I**) Western blots of the phosphorylated CDK2 (CDK2pT160), CDK4 (CDK4pT172) and CDK6 (CDK6pY13) showed that the activated CDK2, 4 and 6 were not significantly changed by either loss of PC2 or rapamycin treatment. (**J**-**L**) Normalized quantitative analyses using the densitometry values from the western blots in **I**. M: months; Bars: 60 μm in **A**-**B**.

**Figure S6.** Rapamycin suppresses mTORC1 downstream indictors: phospho-S6K1 (S6K1pT389), phospho-S6rp (S6rppS235/236), phospho-4E-BP1 (4E-BP1pS65) and phospho-eIF4E (eIF4EpS209) in a dose-dependent manner. (**A**-**D**) Representative western blots of cell lysates with and without PC2 (D3 and E8 cells) showed that S6K1pT389, S6rppS235/236, 4E-BP1pS65 and eIF4EpS209 were significantly more expressed in *Pkd2*-null (E8) cells than in its material control cells (D3). However, both D3 and E8 cells were suppressed in rapamycin dose-dependent manner. (**E**-**H**) Normalized quantitative analyses using the densitometry values from the western blots for these indicators (**A**-**D**).

**Figure S7.** Lacking of PC2 downregulates PC1 expression *in vivo* and *in vitro*. (**A**) Duplicated lysates from 2-month-old *Pkd2*^f3/f3^ kidneys with and without *Vil*-Cre transgene were used to perform western blot analysis using an anti-PC1 polyclonal antibody (mPkd1-Np). Immunoreactivity was weakly observed in the *Vil*-Cre;*Pkd2*^f3/f3^ kidneys, indicating that the lacking PC2 significantly decreases PC1 expression in the disease kidneys. (**B**) Using the same polyclonal antibody, blots of protein lysates from null-*Pkd2* E8 cells and its maternally derived D3 cells (*Pkd2*^+/-^) were positive for immunoreactivity in D3 cells but not E8 cells, suggesting that PC2 loss also downregulates PC1 expression *in vitro*. A *Pkd1*-null cell line (KE1) was used for negative control, and anti-α-tubulin antibody was used to control for protein loading. (**C**-**D**) Normalized quantitative analyses of densitometry values from the 2-month-old *Pkd2*^f3/f3^ kidneys with and without *Vil*-Cre transgene, and *Pkd2*-null cells (E8) and its maternal derived *Pkd2* heterozygous cells (D3), and *Pkd1*-null cells (KE1) were shown (*P* < 0.01). (**E**) Using the anti-PC2 (hPKD2-Cp) and -PC1 (mPkd1-Np) polyclonal antibodies, IF staining (arrows) with mPKD1-Np showed significantly decreased PC1 expression in the *Vil*-Cre;*Pkd2*^f3/f3^ kidneys (Ea *vs* Eb). An anti-PC2 polyclonal antibody hPKD2-Cp was used to be control (Ec *vs* Ed). (**F**) Similar results were observed in IHC staining (arrows) with the same panel antibodies, further validating that the loss of PC2 downregulates PC1 expression *in vivo*. cy=cyst; Bar = 50 μm in **E**-**F**.

**Figure S8.** Rapamycin suppresses the upregulated RB/E2F pathway in the kidneys of 4-month-old *Vil*-Cre;*Pkd2*^f3/f3^ mice with or without Protocol II treatment. (**A**) Duplicate protein lysates from control (*Pkd2*^f3/f3^) kidneys and from rapamycin-treated and placebo-treated *Vil*-Cre;*Pkd2*^f3/f3^ kidneys were immunoblotted with antibodies against phospho-RB (RBpS780)/total RB (t-RB) and E2F; β-actin was used as a protein loading control. (**B**-**C**) Normalized quantitative analyses were performed using densitometry values from the western blots showed in (**A**). Statistical analysis indicated that compared to DMSO placebo, abnormally upregulated elevation of phospho-RB and E2F in *Vil*-Cre;*Pkd2*^f3/f3^ mice was significantly suppressed by rapamycin treatment.

**References**

**Kim I, Fu Y, Hui K, Moeckel G, Mai W, Li C, Liang D, Zhao P, Ma J, Chen XZ, George AL, Jr., Coffey RJ, Feng ZP, Wu G.** Fibrocystin/polyductin modulates renal tubular formation by regulating polycystin-2 expression and function. *Journal of the American Society of Nephrology : JASN*. 2008; 19: 455-68.

**Liu H, Li Y, Li A, Chen Y, Sun C, Ding Z, Wu G.** [Preparation and characterization of a polyclonal antibody against mouse NH2;-terminal domain of polycystin-1]. *Xi Bao Yu Fen Zi Mian Yi Xue Za Zhi*. 2013; 29: 723-8.
